# Analysis of microRNA and Gene Expression Profiles in Multiple Sclerosis: Integrating Interaction Data to Uncover Regulatory Mechanisms

**DOI:** 10.1038/srep34512

**Published:** 2016-10-03

**Authors:** Sherry Freiesleben, Michael Hecker, Uwe Klaus Zettl, Georg Fuellen, Leila Taher

**Affiliations:** 1 Department of Systems Biology and Bioinformatics, University of Rostock, Rostock, Germany; 2 Institute for Biostatistics and Informatics in Medicine and Ageing Research, University of Rostock, Rostock, Germany; 3 Department of Neurology, Division of Neuroimmunology, University of Rostock, Rostock, Germany; 4 Interdisciplinary Faculty, University of Rostock, Rostock, Germany; 5 Division of Bioinformatics, Department of Biology, Friedrich-Alexander Universität Erlangen-Nürnberg, Erlangen, Germany

## Abstract

MicroRNAs (miRNAs) have been reported to contribute to the pathophysiology of multiple sclerosis (MS), an inflammatory disorder of the central nervous system. Here, we propose a new consensus-based strategy to analyse and integrate miRNA and gene expression data in MS as well as other publically available data to gain a deeper understanding of the role of miRNAs in MS and to overcome the challenges posed by studies with limited patient sample sizes. We processed and analysed microarray datasets, and compared the expression of genes and miRNAs in the blood of MS patients and controls. We then used our consensus and integration approach to construct two molecular networks dysregulated in MS: a miRNA- and a gene-based network. We identified 18 differentially expressed (DE) miRNAs and 128 DE genes that may contribute to the regulatory alterations behind MS. The miRNAs were linked to immunological and neurological pathways, and we exposed let-7b-5p and miR-345-5p as promising blood-derived disease biomarkers in MS. The results suggest that DE miRNAs are more informative than DE genes in uncovering pathways potentially involved in MS. Our findings provide novel insights into the regulatory mechanisms and networks underlying MS.

Multiple sclerosis (MS) is one of the most common neurological disorders in young adults and the aetiology of this chronic inflammatory disorder of the central nervous system (CNS) still remains largely unknown. Although many advances regarding MS treatments have been made, there is still no cure. MS is characterized by dysregulated immune mechanisms and seems to develop in genetically susceptible subjects as a result of environmental exposures^1^. The disease manifests as acute focal inflammatory demyelination with incomplete remyelination and axonal loss, which gradually engender multifocal sclerotic plaques in the CNS white matter^2^. These plaques in turn give rise to various cognitive and functional impairments. Several epidemiological and gene expression studies have been conducted in order to elucidate the underlying processes of this disease, and microRNAs (miRNAs), a class of non-coding RNAs, have recently been reported to play a role in the development and progression of MS^3^.

Mature miRNAs are single-stranded endogenous RNAs approximately 22 nucleotides in length that have the ability to posttranscriptionally regulate target messenger RNAs (mRNAs). They bind to the 3′untranslated region of their target mRNAs and translationally repress them or allow for their deadenylation and consequent degradation. It has been shown that the expression of more than 60% of mammalian protein-coding genes is under the control of these small RNAs and that a single miRNA may regulate hundreds of mRNA targets^4^. miRNAs partake in diverse biological processes such as in modulating the immune system and neuroinflammation^5^. They are present in stable form in human blood and plasma, and their expression profiles can be easily investigated, making them ideal MS biomarker candidates^6^. Indeed, a number of miRNA expression profile studies have compared peripheral blood constituents of MS patients to that of healthy controls (HCs), reporting a large number of differentially expressed (DE) miRNAs, as will be detailed below.

Much effort has been devoted to integrating and analysing high-throughput expression and interaction data with the aim of understanding basic principles of human biology and disease. For instance, Gerstein *et al*. constructed a regulatory meta-network by hierarchically organizing the genomic binding information of 119 transcription-related factors derived from the ENCODE project and merging this information with other information, including miRNA regulation^7^. This constituted the first detailed analysis of how regulatory information is organized in human. More specifically, Satoh *et al*. constructed molecular networks from proteomic profiling data derived from MS brain lesions and analysed these networks using four different pathway analysis tools, thereby underlining the relevance of extracellular matrix-mediated focal adhesion and integrin signalling in the development of chronic MS lesions^8^. Riveros *et al*. investigated whole-blood gene expression data of MS patients using a variety of computational methods including transcription factor binding motif (TFBM) overrepresentation analysis and functional profiling, and uncovered a network of transcription factors (TFs) that potentially dysregulate several genes in MS^9^. Similarly, Liu *et al*. created a molecular network based on differentially coexpressed TFs and genes in peripheral blood mononuclear cells (PBMC) of MS patients and performed pathway enrichment analyses to discover regulatory relationships between TFs and target genes^10^. In contrast to the three previously described studies, more recent studies took miRNAs into account when constructing MS-associated molecular networks. Nevertheless, non-overlapping panels of DE miRNAs resulted, possibly because these studies were limited in that they comprised small patient sample sizes, using different high-throughput technologies, or dealing with patients already receiving immunomodulatory treatment. Following microarray analysis of miRNAs and genes in PBMC of MS patients undergoing interferon-beta (IFN-β) treatment, Hecker *et al*. assembled an interaction network of IFN-β-responsive miRNAs and genes using several miRNA target databases^11^. Likewise, Jernås *et al*. generated an interaction network between DE miRNAs and genes in T cells of IFN-β treated MS patients using computationally predicted miRNA targets^12^. Another study by Angerstein *et al*. introduced an approach to construct molecular networks by integrating dysregulated miRNAs in MS, which were uncovered in various studies, and miRNA targets from target gene prediction databases^13^.

Most of the aforementioned studies were conducted in small patient cohorts without technical replicates and independent validation^3^,^14^. It is thus likely that some of the findings are false positives. Beside small patient cohort sizes, these studies were performed using different samples or tissues (e.g., peripheral blood or monocyte), different technological microarray platforms, and different statistical methods to analyse the data. Consequently, little overlap in DE miRNAs can be observed between the various studies in MS^3^,^14^. Consensus methods are commonly used in medicine to define levels of agreement on conflicting data^15^. Hence, a consensus approach based on several expression profile studies is likely to reduce the finding of false positives and to improve the accuracy in identifying genes and miRNAs relevant in MS. In this study, we developed a new consensus-based method to analyse and integrate microarray expression data and other publically available data to gain a deeper understanding of the mechanistic impact of miRNAs in MS and to overcome the challenges posed by small studies. We created two regulatory networks, a miRNA- and a gene-based network, and identified 18 DE miRNAs and 128 DE genes that may contribute to the regulatory alterations behind this inflammatory disease. Of the 18 miRNAs, let-7b-5p and miR-345-5p are the most promising biomarkers. We also show that DE miRNAs are more powerful than DE genes in uncovering pathways potentially involved in MS.

## Results

### miRNA-Based Network

#### Differential MicroRNA Expression in MS

**Figure 1:**
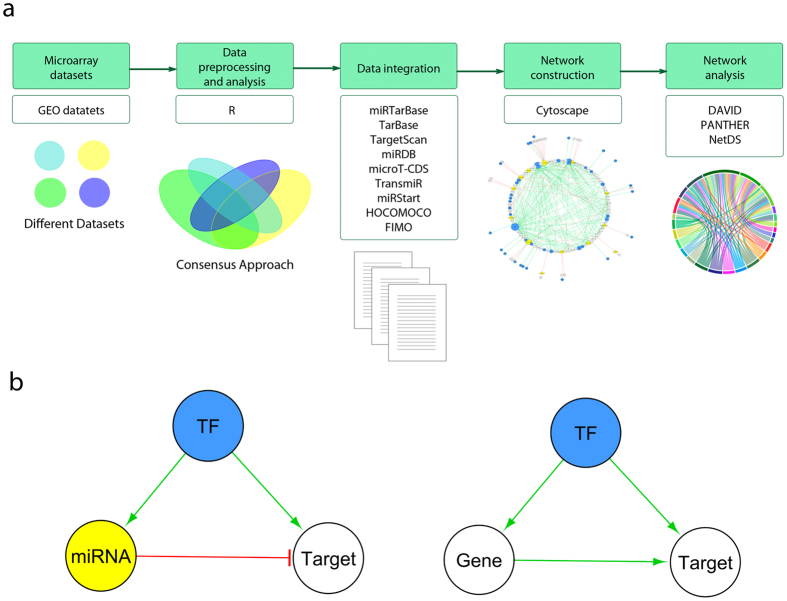
Workflow and general characteristics of the networks in this study. (**a**) Bioinformatics workflow, illustrating the tools and databases employed to uncover the molecules and interactions in the multiple sclerosis (MS)-associated gene- and microRNA (miRNA)-based regulatory networks. (**b**) General configuration of the miRNA- (left) and gene-based (right) networks. The blue nodes represent transcription factors (TFs), the yellow node represents a miRNA, and the white nodes represent molecules that are neither TFs nor miRNAs. The green edges represent activating interactions, whereas the red one represents an inhibitory interaction.

In order to obtain a list of miRNAs involved in MS, we preprocessed and analysed four miRNA microarray datasets (Table 1, Fig. 1). When comparing the miRNA expression levels in the blood of MS patients and HCs, we found a total of 269, 71, 398, and 83 DE miRNAs (t-test p-value ≤ 0.05) in the datasets GSE17846^16^, GSE21079^17^, GSE31568^18^, and GSE39643^19^, respectively, and uncovered 39 miRNAs that were significantly DE (p-value < 0.05) in at least 3 of the 4 datasets (Supplementary Fig. S1). A permutation test suggested that the 39 DE miRNAs are indeed relevant in MS (p-value < 0.002). We next took the direction in which the DE miRNAs were dysregulated into consideration. We thereby identified 18 DE miRNAs that were significantly DE and consistently expressed either at higher or at lower levels in MS in at least 3 of the 4 datasets (Table 2). A second permutation test conferred additional evidence supporting the implication of these 18 DE miRNAs in MS (p-value < 0.002). Out of these 18 candidates for the miRNA-based network, let-7b-5p and miR-345-5p were the only DE miRNAs differentially expressed in the same direction in all four datasets. The average fold-changes of let-7b-5p and miR-345-5p were 1.81 and 1.26 in MS patients compared to HCs, respectively. Hence, let-7b-5p and miR-345-5p are promising blood-derived biomarkers of MS.

#### MicroRNA Targets

We next determined validated and predicted protein-coding gene targets of the 18 DE miRNAs. Using miRTarBase^20^ and TarBase^21^, databases containing experimentally validated miRNA-target interactions, we uncovered 58 validated miRNA-target pairs (Supplementary Table S1). Additionally, we found 21 predicted miRNA-target pairs using a combination of the databases TargetScan^22^, miRDB^23^, and microT-CDS^24^ (Supplementary Table S2). These three databases contain computationally predicted miRNA-target interactions. There was no overlap between the validated and predicted miRNA-target pairs. Thus, we identified 79 miRNA-target interactions in total. Out of the 18 DE miRNAs considered, only 13 had predicted or validated targets (Table 2). We therefore added these 13 miRNAs and their associated targets to the miRNA-based network and excluded the remaining five DE miRNAs since we were interested in exposing interactions and pathways involving MS-associated miRNAs.

#### Regulation by Transcription Factors

We determined transcription factors (TFs) that regulate the 13 DE miRNAs and/or their targets. Using TransmiR^25^, an experimentally supported TF-miRNA regulatory relationship database, we identified 12 validated TF-miRNA interactions (Table 3). Three TFs were part of four feedback loops (FBLs) with miRNAs (Table 3). These TFs and miRNAs include ESR1, SRSF1, LIN28A, let-7b-5p, let-7g-5p, and miR-221-3p. The resulting miRNA-TF interactions were added as miRNA-target interactions, thereby increasing the number of miRNA-target interactions from 79 to 82 (LIN28A repression by let-7b-5p has been identified in both analyses). We also used FIMO^26^, a software tool for scanning DNA sequences with motifs, in combination with HOCOMOCO^27^, a hand-curated collection of transcription factor binding site (TFBS) motifs, and determined 25 predicted TF-miRNA interaction pairs (Supplementary Table S3) and 190 predicted TF-protein-coding gene interactions, that is, TFs targeting miRNA targets (Supplementary Table S4).

#### Construction of the MS-Associated miRNA-Based Network

**Figure 2:**
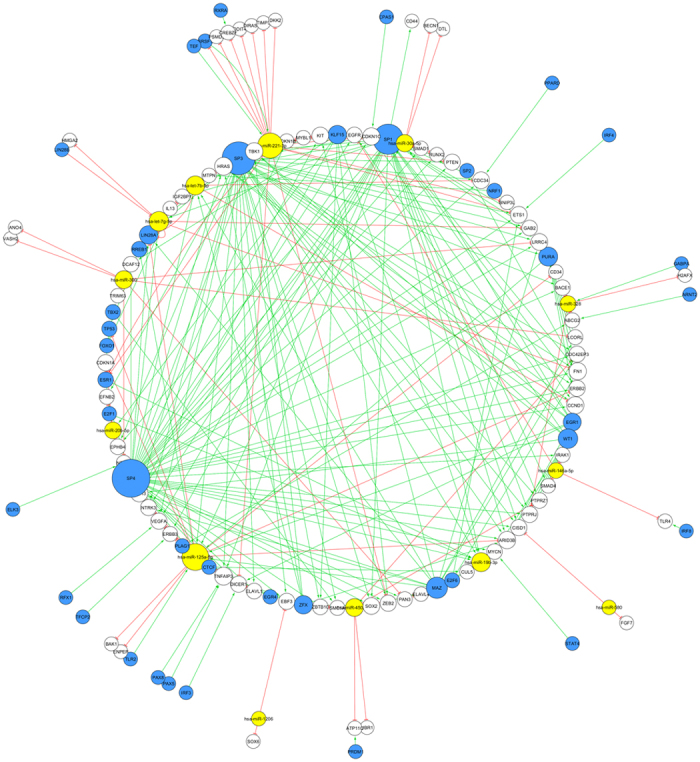
The miRNA-based network dysregulated in multiple sclerosis. This is a circular view of the microRNA (miRNA)-based network. Green edges are activating edges, red ones are inhibiting edges. Yellow nodes represent miRNAs, blue nodes represent transcription factors (TFs), and white ones represent molecules that are not miRNAs or TFs. The size of the nodes is proportional to the degree of the nodes, i.e., the number of incoming and outgoing edges. The three biggest TF nodes are SP4, SP3, and SP1 and the biggest miRNA nodes correspond to miR-125a-5p and miR-221-3p.

Using the above information, we assembled the miRNA-based network (Fig. 2). Our miRNA-based network comprises 130 nodes (13 miRNAs, 78 miRNA targets, and 43 TFs, while 4 TFs were also miRNA targets) and 309 directed edges (82 miRNA-target pairs, 37 TF-miRNA pairs, and 190 TF-gene pairs). Overall, this network indicates that miRNAs are part of a complex regulation system in MS. For instance, miR-125a-5p represses 14 targets and is activated by 10 TFs. This miRNA may therefore be involved in various dysregulated pathways concerning MS.

#### Functional Enrichment Analysis and Subnetworks

**Figure 3:**
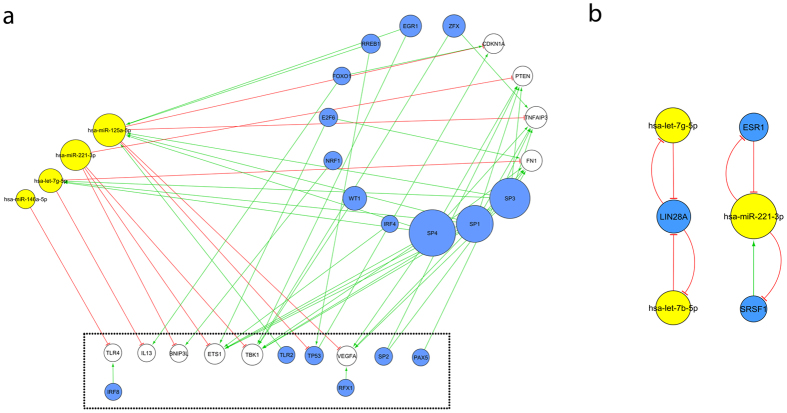
Subnetwork and feedback loops from the microRNA (miRNA)-based network. (**a**) The genes contributing to the enriched gene ontology (GO) term GO:0006955 (immune response) are depicted as nodes in the dashed box. The miRNAs associated to these genes are depicted in yellow on the left and the remaining genes associated to the genes and miRNAs have been circularly arranged on the right. All edges between these nodes (activating edges in green and repressing ones in red) that were present in the full miRNA-based network (Fig. 2) are also present in this subnetwork. The nodes in blue represent transcription factors (TFs). The nodes that are white are nodes that are neither miRNAs nor TFs. The size of the nodes correlates to the degree of the nodes i.e., the number of incoming and outgoing edges, in the full network. The two biggest miRNA nodes correspond to miR-125a-5p and miR-221-3p and repress targets that contribute to the enriched GO term GO:0006955 (immune response). (**b**) The nodes and edges involved in the four feedback loops of the miRNA-based network (Table 3) are depicted. miR-221-3p is also involved in these feedback loops.

We next performed a functional analysis on all the nodes of the miRNA-based network using DAVID^28^. By this means, we discovered 410 significantly enriched terms (FDR ≤ 0.05), of which, 16 gene ontology (GO) terms were immunology-related or neurology-related (Table 4). We inspected the association of the miRNAs in the network with the 16 enriched GO terms in order to associate specific miRNAs to possibly dysregulated pathways in MS. Therefore, for each enriched immunology- and neurology-related GO term, we created a subnetwork using the genes associated with this term as well as the directly interacting, neighbouring, nodes (Fig. 3a, Table 4, Supplementary Figs S2–S14). For example, we found that four miRNAs are present in the subnetwork created from the genes belonging to the enriched GO term GO:0006955 (immune response), indicating that they might be involved in interactions linked to the immune response in MS.

We also categorized the 16 enriched immunology- and neurology-related GO terms into four groups: 1) Innate immune and inflammatory responses; 2) Immune response and immune system development; 3) Immune cells and immune tissue development; and 4) Neuron development and plasticity. miR-125a-5p is present in all subnetworks created from these GO terms, suggesting that this miRNA is crucially implicated in various dysregulated pathways in MS. On the other hand, let-7g-5p, miR-19b-3p, miR-30a-5p, and miR-221-3p are mainly involved in subnetworks created from enriched GO terms corresponding to the second and third category. This indicates that these miRNAs are involved in modulating cells regarding autoimmunity and inflammation in MS as well as in affecting the immune response and immune system development. Additionally, miR-221-3p is associated with all the enriched neurology-related GO terms, providing confidence that this miRNA plays a role in influencing molecular processes relevant to MS. miR-450b-5p is also affiliated to neuron differentiation, neuron development, neuron projection development, and positive regulation of neurogenesis, but not to the regulation of long-term neuronal synaptic plasticity. This finding suggests that miR-450b-5p may be implicated in the early stages of CNS development instead of later stages. In the context of MS, this miRNA may be thus particularly important in the relapsing-remitting phase of the disease.

Finally, a pathway enrichment analysis of the miRNA-based network nodes using PANTHER^29^ revealed that the nodes in the network are part of enriched immunological pathways (Table 5) such as the toll receptor signalling pathway (FDR = 7.6 × 10^−5^) and the interleukin signalling pathway (FDR = 1.1 × 10^−3^). Taken together, these results strongly support the hypothesis that miRNAs are involved in key dysregulated immunological and neurological pathways in MS.

#### Network Analysis

We next carried out a FBL and feed forward loop (FFL) analysis since it has been shown that miRNAs participating in these types of loops act as regulatory switches giving rise to distinct cellular states^30^. Our FBL and FFL analysis revealed the presence of four FBLs (one negative and three positive FBLs) as depicted in Fig 3b. let-7b-5p, let-7g-5p, and miR-221-3p are involved in either one of these four FBLs as well as in subnetworks created from the enriched immunology- and neurology-related GO terms. We further unveiled a total of 107 FFLs (42 coherent and 65 incoherent) embedded within the miRNA-based network. Seven miRNAs, let-7b-5p, let-7g-5p, miR-19b-3p, miR-20b-5p, miR-30a-5p, miR-125a-5p, and miR-221-3p, were involved in these FFLs. Except for miR-20b-5p, all these miRNAs were also involved in the subnetworks created from the enriched immunology- and neurology-related GO terms (Table 4). As let-7b-5p, let-7g-5p, and miR-221-3p participate in both FFLs and FBLs, these miRNAs have the potential to greatly influence the fate of cells in MS.

### Protein-coding gene-Based Network

#### Differential protein-coding gene Expression in MS

In order to obtain a list of protein-coding genes relevant in MS, we preprocessed and analysed four microarray datasets (Table 1). For simplicity, we will refer to these protein-coding genes simply as genes from this point on. We found a total of 431, 6099, 786, and 3717 DE genes (t-test p-value ≤ 0.05) in the datasets GSE17048^9^, GSE21942^14^, GSE41890^31^, and GSE43591^12^, respectively, and 267 genes that were significantly DE in MS compared with controls (p-value ≤ 0.05) in at least 3 of the 4 datasets (Supplementary Fig. S15, p-value < 0.0002), suggesting that they are involved in MS. We next took the direction in which the genes were DE into consideration. We thereby identified 128 genes that were consistently expressed at significantly higher or lower levels in the blood of MS patients than of HCs in at least 3 of the 4 datasets (Supplementary Table S5, p-value < 0.0002). BEX1 and BEX2 were upregulated and PALLD and ZNF264 were downregulated in MS in all four datasets.

#### Gene Regulatory Targets

Similar to the steps taken in constructing the miRNA-based network, we uncovered validated and predicted DE gene targets to be included in the gene-based network. For this purpose, we made use of TransmiR and revealed that AKT3 activates both miR-22-3p and miR-22-5p, two miRNAs that are not present in the miRNA-based network. We therefore added these interactions to the gene-based network. Furthermore, we searched DE genes that were also TFs in the HOCOMOCO databases in order to confidently ascertain their putative targets. DDIT3 was the only DE gene present in HOCOMOCO, and we identified 91 predicted targets of DDIT3. We included these 91 interactions in the gene-based network, and therefore identified a total of 93 interactions between DE genes and validated and predicted targets.

#### Regulation by Transcription Factors

We uncovered predicted TF-target interactions using the 128 DE genes and the 93 DE gene targets. We thereby determined 315 predicted TF and DE gene interaction pairs, and 233 potential interactions between TFs in HOCOMOCO and DE gene targets. Thirteen of the latter interactions were related to DDIT3, as described in the previous section. Thus, 220 additional interactions could be revealed. Using miR-22-3p and miR-22-5p, we further found 8 predicted TF-miRNA interaction pairs. In total, we gathered 543 interactions for the gene-based network.

#### Construction of the MS-Associated Gene-Based Network

**Figure 4:**
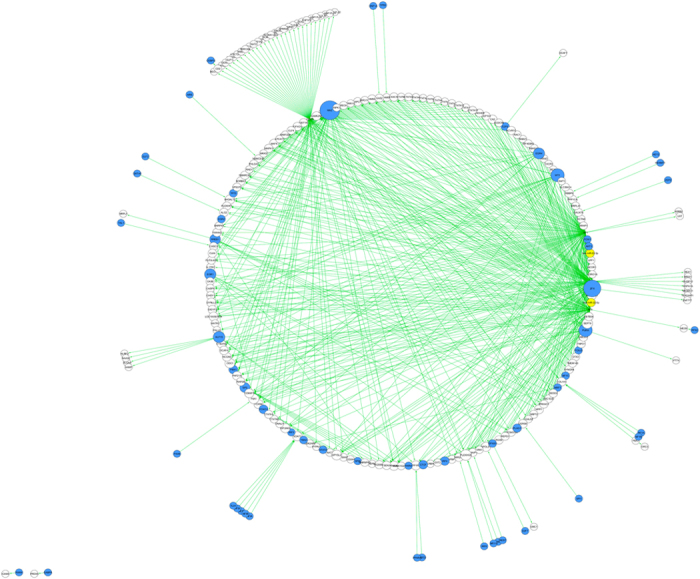
The protein-coding gene-based network dysregulated in multiple sclerosis. This is a circular view of the protein-coding gene-based network. Green edges are activating edges and red ones, inhibiting edges, are not present in this network. Yellow nodes represent miRNAs, blue nodes represent transcription factors (TFs), and white ones represent molecules that are not miRNAs or TFs. The size of a node is proportional to the degree of the node i.e., the number of incoming and outgoing edges. Unlike the miRNA-based network (Fig. 2), the largest TF nodes correspond to MAZ and ZFX. The only miRNAs present in this network are miR-22-3p and miR-22-5p which are both not present in the miRNA-based network.

We next assembled the gene-based network using the 93 interactions between DE genes and their targets, the 315 interactions between TFs and DE genes, and the 228 interactions between TFs and DE gene targets. Therefore, the final gene-based network comprised a total of 636 interactions and 244 nodes (Fig. 4). A subset of 92 of the original 128 DE genes is present in the gene-based network. AKT3 and DDIT3 are the only DE genes that are activated by TFs and that act as TFs.

#### Functional Enrichment Analysis

A functional analysis using DAVID with the network nodes revealed six significantly enriched immunology-related terms (Table 6, FDR ≤ 0.05). AKT3 is not present in these enriched terms therefore we could not link miR-22-3p or miR-22-5p to enriched terms. This finding suggests that the gene-based network is indicative of some immunological mechanisms but not for neurological abnormalities involved in MS.

We next carried out a pathway enrichment analysis of the gene-based network nodes using PANTHER to detect affected pathways in MS. The only significantly enriched pathway was the p53 pathway by glucose deprivation (FDR = 0.001). This finding suggests that the gene-based network does not reflect specific regulation events related to MS.

#### Network Analysis

We did not identify any FBLs, however we discovered the presence of 126 coherent FFLs. This demonstrates that the DE genes may be involved in regulatory loops influencing MS, however the gene-based network is less indicative of MS-associated processes compared to the miRNA-based network. We conclude that more insights regarding dysregulated pathways in MS can be gained by investigating dysregulated miRNAs instead of genes.

## Discussion

The complex functions of miRNAs, especially in diseases, are still poorly understood. Due to the limited number of public miRNA microarray expression profiles in MS, it is still unclear, which miRNAs play a pivotal role in this chronic disease. In the present study, we made use of publicly available microarray data and databases with the purpose of identifying blood-derived miRNA and mRNA biomarkers as well as molecular interactions that clarify biochemical mechanisms behind MS. To this end, we created a miRNA- and a gene-based network. Our networks differ from previous studies in the literature in that they are based on a consensus of multiple microarray datasets. Based on our networks, we were able to identify pathways potentially involved in MS and generated a list of blood miRNA biomarkers.

Because of the inaccessibility of the nervous system, most MS expression studies involve either post-mortem samples or readily obtainable tissue, in particular blood. In the search for biomarkers, the assumption is that the inflammatory and neurodegenerative processes in the CNS are reflected, at least in part, in peripheral blood cells. Thus, genetic variants altering the expression of MS-relevant miRNAs that are not cell-type specific may lead to changes in multiple cells, including blood cells^32^. In addition, blood-brain barrier dysfunction in MS leads to the pronounced infiltration of immune cells in the brain, facilitating the transport of miRNAs to the site of inflammation. In particular, exosomes have been shown transfer miRNAs between cells, perhaps also from immune cells to glial cells^33^.

The three microarray platforms associated to the miRNA datasets used in this study (Table 1) each contain less than 900 mature miRNAs identifiers, although over 2000 mature human miRNAs are available through the miRBase database (release 19)^34^. This difference in number may hinder the identification of additional miRNAs involved in MS and associated dysregulated pathways. Despite this limitation, our consensus strategy identified 18 miRNAs that account for differences between MS patients and HCs. Eleven of these 18 miRNAs were reported to be significantly DE in at least one of the original studies that generated the four microarray datasets that we used to construct the miRNA-based network (Table 2). The fact that not all 18 miRNAs were found to be significantly DE in at least three of the four original studies can be explained by differences in normalization and analysis methods. The MS relevance of the 18 miRNAs in the consensus is supported by a variety of other independent expression profiling studies (Table 2), suggesting that our approach enables us to overcome replication issues associated with variations in experimental protocols and microarray platforms, and small sample sizes.

We identified two potential MS miRNA biomarkers, let-7b and miR-345, that were significantly upregulated in MS according to all four datasets analysed. miR-345 has also been shown to be overexpressed in CD19^+^ B cells of systemic lupus erythematosus (SLE) patients^35^. In MS, it has been demonstrated that there is a significant increase in the number of CD19^+^ cells in the blood^36^. Therefore, it would be of interest to perform cell type-specific studies to validate miR-345 as a biomarker for the diagnosis and prognosis of MS. Two recent studies did not observe different let-7b levels between individuals with MS and HCs^37^,^38^. This miRNA has however been linked to neurodegeneration; elevated amounts of let-7b were found in the cerebrospinal fluid (CSF) of patients with Alzheimer’s disease^39^. The injection of let-7b into the CSF of mice resulted in neurodegeneration via TLR7 signalling^39^. In turn, it has been shown that TLR7 expression is decreased in PBMCs and monocytes of MS patients compared to HCs, while IFN-β therapy restores TLR7 levels^40^. IFN-β also upregulated let-7b *in vitro* in macrophages and forms a negative FBL with let-7b^41^. Furthermore, IFN-β therapy induced the expression of let-7b in MS patients^11^. Hence the role of let-7b in the context of the treatment of MS with IFN-β should be investigated in more detail.

In our miRNA-based network, miR-125a, miR-221, miR-300, and miR-450b have 14, 18, 8, and 8 targets, respectively. These four miRNAs regulate more targets than the other nine miRNAs in the network in combination. This may be because the sheer amount of information concerning these miRNAs is greater compared to that of the other miRNAs, or because these miRNAs may have a more important role in MS. We did not observe any overlap between these 48 miRNA targets and the 128 DE genes used to construct the gene-based network. This lack of overlap may reflect the facts that miRNAs regulate their targets posttranscriptionally without necessarily degrading their target mRNAs, and that most genes have multiple regulators, and their expression levels are functions of multiple inputs. Furthermore, despite our careful selection, the microarray experiments that form the basis of our miRNA and gene networks were not all performed on the same cell populations. The up-regulation of a miRNA in, for instance, monocytes may not necessarily be strongly correlated with the expression profiles of its target genes in other PBMCs. Likewise, since the miRNA and gene expression datasets are not paired, but rather, truly independent samples, interindividual differences may potentially mask the largely fine-tuning regulatory effects of miRNAs. The availability of paired miRNA and mRNA expression datasets for large patient cohorts should provide additional insights. Ultimately, validating predicted regulatory mechanisms requires experiments with miRNA mimics/inhibitors.

We provided evidence that certain MS-associated miRNAs are involved in neurological processes and may influence components of the immune system. We exposed that miR-125a, which was increased in expression in MS patients compared to HCs, is associated to 16 enriched immunology- and neurological-related GO terms (Table 4). Recently, decreased levels of miR-125a were detected in blood samples of MS patients after natalizumab treatment initiation^42^. Moreover, a microarray analysis by Jernås *et al*. revealed an upregulation of miR-125a in peripheral blood T cells of both IFN-β-treated and untreated MS patients compared to HCs^12^. Comparable to our miRNA-based network, they highlighted that miR-125a targets KLF13 and TNFAIP3^12^. In our network, we included these interactions as well as the induction of miR-125a by TLR2. In SLE, miR-125a was shown to negatively regulate RANTES, an inflammatory chemokine, by targeting and inhibiting KLF13^43^. In addition, miR-125a was described to directly repress TNFAIP3^44^, a negative regulator of NF-κB signalling and inflammation, which is expressed at lower levels in monocytes of relapsing-remitting MS (RRMS) patients compared to HCs^45^. We thus suggest that miR-125a is employed to fine-tune inflammation, and drugs such as IFN-β and natalizumab may influence inflammation by modifying miR-125a levels.

Besides miR-125a, we unveiled three other miRNAs highly connected to immune cells and immune tissue development: let-7g, miR-19b, and miR-30a (Table 4). In accordance with our study, let-7g was previously found to be upregulated in MS patients^46^ and to be DE between secondary progressive MS (SPMS) patients and HCs^47^. let-7g levels in circulating blood leukocytes are, however, significantly lower after acute inflammation^48^. Therefore, changing let-7g levels may be employed to regulate inflammation. We also observed that miR-19b is upregulated in MS patients and it was likewise reported to be upregulated in regulatory T cells of RRMS patients compared to HCs^49^. On the other hand, in natalizumab-treated MS patients, miR-19b levels were lower compared to untreated RRMS patients^50^. Since we uncovered that miR-19b is involved in coherent and incoherent FFLs and because it is associated to leukocyte differentiation (Table 4, Supplementary Fig. S6), miR-19b may affect the differentiation of diverse immune cell types. We additionally found that miR-30 was upregulated in the blood of MS patients compared to HCs. This miRNA is upregulated in inactive MS lesions compared to normal brain tissue^51^, and it was already reported that it is significantly dysregulated in the blood in RRMS patients compared to HCs^52^. This is in line with another study, which revealed significantly altered levels of miR-30a in MS during remission^53^. The overexpression of this miRNA in B cells was shown to cause an increase in B cell proliferation and the production of IgG antibodies^54^. It is therefore suggested that miR-30a plays an important role in B cell hyperactivity. Our results also support this role of miR-30a because it was associated to leukocyte differentiation (Table 4, Supplementary Fig. S6) and it was involved in FFLs.

Our analysis exposed higher levels of miR-221-3p in in the blood of MS patients compared to HCs and revealed that miR-221 was associated with GO terms concerning immune system development, immune response, neuron development, and neuron plasticity. We also showed that miR-221 was involved in many coherent FFLs. In cell type-specific studies, MS patients were found to exhibit higher levels of this miRNA in regulatory T cells^49^ and lower levels were in B cells^50^ compared to HCs. Therefore, miR-221 expression may impact the development of certain immune cells which can influence neurogenesis in MS.

Finally, another miRNA that potentially participates in neurogenesis and neurodifferentiation is miR-450b. We found it to be downregulated in MS and it was associated with enriched neurology-related GO terms. However it has not yet been connected to neurological disorders. PTPRZ1, a miR-450b target, is expressed in remyeliating oligodendrocytes in MS lesions^55^. SOX2, another miR-450b target, is expressed by immature Schwann cells and inhibits Schwann cell differentiation and myelination^56^. Based on these findings, the impact of miR-450b on myelination in MS should be examined in future studies.

Compared to our miRNA-based network, our gene-based network did not shed light on many potential regulatory events behind MS. Despite finding four candidate genes (BEX1, BEX2, PALLD, and ZNF264) that were consistently either up- or downregulated in the blood of MS patients in all four datasets, these DE genes were not associated to enriched immunology- or neurology-related terms. Although their gene products have been shown to be involved in neurology^57^,^58^,^59^, these DE genes were not part of FFLs. Even though the gene-based network is slightly enriched in immune-related terms (Table 6), it does not reflect regulatory mechanisms concerning specific aspects of MS. This discrepancy in the results suggests that posttranscriptional events may play a greater role than previously anticipated in dysregulating regulatory mechanisms in this disease. A dysregulated miRNA may have a greater impact on the development of MS compared to a dysregulated protein-coding gene since a miRNA may regulate hundreds of mRNA targets^60^. Genetic variants such as single nucleotide polymorphisms can lead to the aberrant expression of miRNAs and increase the risk of developing certain diseases^61^,^62^.

In conclusion, we presented a consensus-based method to analyse and integrate gene and miRNA expression data as well as other publically available data. Our results revealed that DE miRNAs are more informative than DE genes when uncovering potential molecular pathways involved in MS. We argued that, among others, let-7b-5p and miR-345-5p might be the most promising blood-derived miRNA biomarkers in MS.

## Methods

In this study, we emphasized on identifying candidate miRNA and gene biomarkers that are DE in multiple MS microarray datasets. For this purpose, we developed a consensus and database integration approach to construct a miRNA- and a gene-based disease-associated regulatory network. Figure 1 depicts the workflow used for this study as well as the general characteristics of the networks. We assembled these networks with the aim of uncovering interactions between miRNAs and genes potentially implicated in the onset and progression of MS.

### Microarray Data Preprocessing

We downloaded publically available microarray datasets, containing raw or normalized data, together with the corresponding platform specifications from the Gene Expression Omnibus (GEO) database (Table 1). We excluded studies in which patients were undergoing treatment or in which samples were not blood-derived. The dataset containing raw data, GSE41890, was robust multiarray average RMA-normalized using the Affy package^63^ in R (version 2.12.1). This dataset delivers gene expression levels in the blood of 22 MS patients measured at two different time points. For the analysis of this dataset, we only use one of the two samples per patient. In datasets containing technical replicates, we averaged the expression data of the replicate microarrays. All clinical subtypes of MS were included in this analysis because gene expression differences between the subtypes are comparatively minor^64^. In case of the miRNA microarray datasets, we converted the assay identifiers to current miRNA names provided by the miRBase database (release 19)^34^. Only the data of assay identifiers with one-to-one relationship to miRNA names were included in the analysis. In case of the gene expression microarray datasets, we converted probe identifiers to official gene symbols. Data of identifiers that could not be converted were excluded. For each sample, we averaged the expression data of identifiers that were assigned to identical gene symbols.

### Differential Expression Analysis and Consensus Approach

For each dataset and each gene and miRNA, we performed t-tests comparing the data of HCs and MS patients. For each miRNA and each gene, we also calculated the fold-change, that is, the ratio of the average expression in MS patients versus the average expression in HCs. A miRNA or gene is upregulated if this ratio is greater than one, downregulated if smaller than one, and unchanged if equal to one. miRNAs that were DE with p-value ≤ 0.05 in at least 3 of the 4 miRNA datasets and that were consistently significantly up- or downregulated in MS were selected for the further functional and interaction analyses. For this study, we used the following two criteria to define consensus: 1) A transcript has to be differentially expressed in three out of the four experiments corresponding to the gene or miRNA datasets in Table 1 and 2) the change in expression in a transcript has to be in the same direction (up or downregulation) in the three or four experiments. Similarly, we selected genes for the gene-based network that were DE with p-value ≤ 0.05 in at least 3 of the 4 gene expression datasets and that were DE in the same direction in these data.

### Permutation Test of Differential Expression Consensus

We performed permutation tests in order to demonstrate that our selected DE miRNAs and genes are relevant in MS and that they are not the result of random selection. Firstly, for each miRNA dataset, we randomly selected a number of miRNAs equal to the number of actually DE miRNAs. We then determined the number of miRNAs that were common in at least 3 of the 4 miRNA datasets. We repeated this process 5000 times and, in this way, we calculated a p-value for our actually selected DE miRNAs without taking up- or downregulation into consideration. For a second permutation test, we took into account the direction of DE miRNAs and genes. Similar to the first test, we randomly selected a number of miRNAs corresponding to the number of actually DE miRNAs for each dataset. We then determined the number of miRNAs that were common in at least 3 of the 4 miRNA datasets and consistently expressed either at higher or at lower levels in the MS patient group compared with HCs. We repeated this process 5000 times to calculate a p-value for the actually selected DE miRNAs that were common in at least 3 datasets and always dysregulated in MS in the same direction. We correspondingly carried out these two tests for the selected DE genes to calculate respective p-values.

### MicroRNA Target Analysis

Five databases comprising miRNA targets were used. Of these, miRTarBase (release 3.5) and TarBase (release 6.0) contain validated miRNA targets^20^,^21^. Within TarBase, we only retained validated miRNA targets that have been confirmed via reporter gene assays. All other validation methods (e.g., microarray and sequencing) were discarded because these methods indicate a correlation of expression between miRNAs and their potential targets rather than causation, i.e., miRNAs binding to their targets thus causing a decreased expression of their targets. Likewise in miRTarBase, we discarded all verified targets for which the miRNA-target interaction was classified as weak. In contrast, TargetScan (release 6.2), miRDB (release 4.0), and microT-CDS (release 5.0) consist of predicted miRNA targets^22^,^23^,^24^. In TargetScan, targets with a context score smaller or equal to −0.19 were retained. In microT-CDS, targets with a miTG score greater or equal to 0.993 were kept. Predicted targets from miRDB with a score greater or equal to 84 were also kept. We chose these rather strict cut-offs in an attempt to reduce the number of false positives. Finally, DE miRNA-target gene pairs that were common to all 3 databases were extracted. In the miRNA-based network, we did not visualize DE miRNAs without a verified or predicted target.

### Transcription Factor Target Analysis

Validated TF-miRNA interactions and their regulation (activation or repression) were exported from the TransmiR database (release 1.2)^25^. Predicted TF-miRNA and TF-gene interactions were on the other hand determined by first retrieving the promoter sequences of all previously identified miRNAs and genes. We defined the promoter region as a 2 kbp sequence starting 1.5 kbp upstream of the transcription start site (TSS) and ending 0.5 kbp downstream of the TSS. We obtained miRNA TSS using miRStart (release July 21, 2010) and gene TSS using RefGene^65^,^66^. We afterwards ran FIMO, a motif search tool of the MEME suite, together with HOCOMOCO, a database containing hand-curated transcription factor binding site (TFBS) models, on the corresponding repeat-masked sequences to identify TFs that potentially bind to the promoter regions^26^,^27^. TF-miRNA and TF-gene interaction predictions with p-value ≤ 0.05 were retained. We kept the top 1% of these predicted interactions and deleted all duplicate interactions.

### Regulatory Network Construction

In the miRNA-based network, we included DE miRNAs, their targets, TFs regulating these miRNAs and their targets as well as the type of interaction between these molecules. We assumed that all miRNAs repress their targets, unless otherwise indicated in TransmiR. miRNA target activation is possible but remains a rare event^67^. We also assumed that TFs activate their targets, unless otherwise indicated in TransmiR. The gene-based network was created in a similar fashion. The networks were constructed and visualized using Cytoscape^68^ (version 3.2.0). We employed NetDS, a plugin for Cytoscape, to uncover FFLs and FBLs that contribute to the complexity of the regulatory networks in MS^69^. The Cytoscape session files are available from the corresponding authors upon request.

### Functional Enrichment Analysis

In order to assess which functionally related genes (e.g., as defined by GO terms) are predominantly represented in either the miRNA- or gene-based network, we performed a functional annotation analysis using DAVID (release 6.7)^28^. We adjusted the p-value of the enriched terms for multiple testing using the Benjamini and Hochberg (BH) method^70^. We also carried out a pathway overrepresentation test in PANTHER^29^ (version 10.0) and adjusted the p-value of the enriched pathways using the BH procedure.

### Subnetwork Creation

We created subnetworks based on the enriched GO terms found using DAVID and the nodes in the miRNA-based network. From our complete miRNA-based network, we selected the genes associated to an enriched GO term as well as all their neighbouring nodes in order to associate miRNAs to specific GO terms. We also selected all edges between the genes and their first neighbouring nodes when creating the subnetworks.

## Additional Information

**How to cite this article**: Freiesleben, S. *et al*. Analysis of microRNA and Gene Expression Profiles in Multiple Sclerosis: Integrating Interaction Data to Uncover Regulatory Mechanisms. *Sci. Rep*. **6**, 34512; doi: 10.1038/srep34512 (2016).

## Supplementary Material

Supplementary Information

## Figures and Tables

**Table 1:** Microarray datasets used for the differential expression analysis.

GEO dataset	Data	Platform	Controls	MS	Tissue	Reference
microRNAs						
GSE17846	Normalized	GPL9040	21	20	Peripheral blood	16
GSE21079	Normalized	GPL8178	37	59	Peripheral blood	17
GSE31568	Normalized	GPL9040	70	23	Peripheral blood	18
GSE39643	Normalized	GPL15847	8	8	Blood-derived monocytes	19
Genes						
GSE17048	Normalized	GPL26947	45	99	Peripheral blood	9
GSE21942	Normalized	GPL570	15	12	PBMC	14
GSE41890	Raw	GPL6244	24	22	Peripheral blood leukocytes	31
GSE43591	Normalized	GPL570	10	10	Peripheral blood	12

GEO dataset: Gene Expression Omnibus dataset (series) are represented by a series accession number beginning with the letters GSE; Platform: a platform provides the physical setup of an assay such as an array and is linked to a GEO platform accession number beginning with the letters GPL; Controls: control samples; MS: number of multiple sclerosis patient samples; PBMC: peripheral blood mononuclear cells.

**Table 2:** Differentially expressed microRNAs in our study and in other multiple sclerosis studies.

microRNA	Regulation	DE consensus	DE in extra miRNA studies in MS
**let-7b-5p**	up	16, 17, 18, 19	11,19
**let-7g-5p**	up	16,18,19	17,46,47
**miR-19b-3p**	up	16,18,19	19,49,50
**miR-20b-5p**	down	16, 17, 18	16,17,47,51,52,71
**miR-30a-5p**	up	16,18,19	16,51, 52, 53
**miR-125a-5p**	up	16, 17, 18	12,42,72,73
**miR-146a-5p**	up	16,18,19	19,51,74, 75, 76, 77
miR-186-5p	up	16,18,19	16
**miR-221-3p**	up	16,18,19	19,47
**miR-300**	down	16,18,19	—
**miR-328**	up	16, 17, 18	16,51,53,73
miR-345-5p	up	16, 17, 18, 19	—
miR-363-3p	down	16, 17, 18	46,50,73
miR-379-5p	down	16,18,19	19
**miR-450b-5p**	down	16,18,19	—
**miR-580**	down	16,18,19	—
miR-664a-3p	up	16,18,19	—
**miR-1206**	down	16,18,19	19

Listed under the header “microRNA” are the 18 microRNAs (miRNAs) that were differentially expressed (DE) in our study and that were DE in the same direction in at least three of the four miRNA expression datasets used for this study. A brief description of these miRNA expression datasets can be found in Table 1. “Up” regulated means that a miRNA is expressed at a higher level in multiple sclerosis (MS) patients compared to controls and vice versa for “down” regulation. In the third column, we provide references to the datasets in which we found the miRNAs to be differentially expressed in the same direction in at least three of the four miRNA expression datasets. In the last column, references to additional studies in which these miRNAs are also described as differentially expressed are indicated. miRNA names in bold indicate the 13 miRNAs that were included in the miRNA-based network.

**Table 3:** Transcription factor and microRNA regulation pairs found using TransmiR.

TF	miRNA	Regulation	FBL
E2F1	miR-19b-3p	Activation	
E2F1	miR-20b-5p	Activation	
EGR1	miR-30a-5p	Activation	
EGR1	miR-125a-5p	Activation	
ESR1	miR-19b-3p	Activation	
ESR1	miR-20b-5p	Activation	
ESR1	miR-221-3p	Repression	×
LIN28A	let-7b-5p	Repression	×
LIN28A	let-7g-5p	Repression	×
LIN28B	let-7g-5p	Repression	
SRSF1	miR-221-3p	Activation	×
TLR2	miR-125a-5p	Activation	

Transcription factors (TFs), their microRNA (miRNA) targets and the type of regulation that the TFs exercise are shown. It is also indicated, which TFs and miRNAs mutually regulate each other through feedback loops (FBL), see also Fig. 3b.

**Table 4:** Differentially expressed microRNAs present in the subnetworks.

Term ID	GO term name	let-7b-5p	let-7g-5p	miR-19b-3p	miR-30a-5p	miR-125a-5p	miR-146a-5p	miR-221-3p	miR-450b-5p	miR-1206
1) Innate immune and inflammatory responses										
GO:0002218	Activation of innate immune response					X	X			
GO:0002758	Innate immune response-activating signal transduction					X	X			
GO:0002224	Toll-like receptor signaling pathway					X	X			
GO:0002237	Response to molecule of bacterial origin					X	X			
GO:0006954	Inflammatory response		X		X	X	X			
2) Immune response and immune system development										
GO:0006955	Immune response		X			X	X	X		
GO:0002520	Immune system development		X	X	X	X		X		X
3) Immune cells and immune tissue development										
GO:0002521	Leukocyte differentiation		X	X	X	X		X		
GO:0045321	Leukocyte activation		X	X	X	X	X			
GO:0030099	Myeloid cell differentiation		X	X	X	X		X		X
GO:0048534	Hemopoietic or lymphoid organ development		X	X	X	X		X		X
4) Neuron development and plasticity										
GO:0030182	Neuron differentiation	X				X	X	X		
GO:0050769	Positive regulation of neurogenesis				X	X		X	X	
GO:0048666	Neuron development					X	X	X	X	
GO:0031175	Neuron projection development					X	X	X	X	
GO:0048169	Regulation of long-term neuronal synaptic plasticity	X			X	X		X		

Each row corresponds to an enriched immunology- or neurology-related GO term found by performing a functional enrichment analysis using all nodes in the miRNA-based network dysregulated in MS. Four major GO term categories were distinguished. The respective information was used to create subnetworks (Fig. 3a, Supplementary Figs S2–S14). The presence of MS-associated miRNAs in the different subnetworks is marked by “X”.

**Table 5:** Pathways related to the microRNA-based network.

Pathway	p-value
Toll receptor signaling pathway	7.6 × 10^−5^
Interleukin signaling pathway	0.001
EGF receptor signaling pathway	0.004
PI3 kinase pathway	0.006
p53 pathway feedback loops 2	0.006
CCKR signaling map	0.01
PDGF signaling pathway	0.02
Gonadotropin releasing hormone receptor pathway	0.03
Angiogenesis	0.03

Shown are the enriched pathways found after performing a PANTHER^29^ analysis with all the nodes of the microRNA-based network. The p-values are corrected for multiple testing using the Benjamini-Hochberg^70^ (FDR) method.

**Table 6:** Immunology-related terms associated with the nodes of the gene-based network.

Term name	Genes	p-value
GO:0030099 myeloid cell differentiation	CEBPG, EPAS1, IRF4, IRF8, NCOA6, SP1, SP3, TAL1	0.0005
GO:0048534 hemopoietic or lymphoid organ development	CEBPG, EGR1, EPAS1, IRF4, IRF8, NCOA6, PBX1, SP1, SP3, TAL1, TLX1, TP53	0.001
GO:0002520 immune system development	CEBPG, EGR1, EPAS1, IRF4, IRF8, NCOA6, PBX1 SP1, SP3, TAL1, TLX1, TP53	0.002
PIRSF005710 interferon regulatory factor 3-9	IRF3, IRF4, IRF8	0.006
GO:0042110 T cell activation	CD2, EGR1, ELF4, IRF4, SP3, TP53	0.03
GO:0046649 lymphocyte activation	CD2, CEBPG, EGR1, ELF4, IRF4, SP3, TP53	0.05

Functional terms were tested for enrichment using DAVID^28^ with the 244 nodes of the gene-based network that is dysregulated in multiple sclerosis (Fig. 4). The p-values were corrected for multiple testing using the Benjamini-Hochberg^70^ (FDR) method.
