# Supplementary material for: Analysis of microRNA and Gene Expression Profiles in Multiple Sclerosis: Integrating Interaction Data to Uncover Regulatory Mechanisms
Source: Sci Rep. 2016 Oct 3;6:34512. doi: 10.1038/srep34512 (PMC5046091; doi:10.1038/srep34512)
Supplement: Supplementary Information [file srep34512-s1.pdf]

Analysis of microRNA and Gene Expression Profiles in Multiple Sclerosis: Integrating Interaction Data to Uncover Regulatory Mechanisms

Sherry Freiesleben[1,2], Michael Hecker[3], Uwe Klaus Zettl[3], Georg Fuellen[2,4], Leila Taher[2,5,*]

[1] Department of Systems Biology and Bioinformatics, University of Rostock, Rostock, Germany.

[2] Institute for Biostatistics and Informatics in Medicine and Ageing Research, University of Rostock, Rostock, Germany.

[3] Department of Neurology, Division of Neuroimmunology, University of Rostock, Rostock, Germany.

[4] Interdisciplinary Faculty, University of Rostock, Rostock, Germany.

[5] Division of Bioinformatics, Department of Biology, Friedrich-Alexander Universität Erlangen-Nürnberg, Erlangen, Germany.

* **Corresponding authors:** E-mail: leila.taher@fau.de

Supplementary Tables and Figures

Contents

Supplementary Figure 1 3

Supplementary Figure 2 4

Supplementary Figure 3 5

Supplementary Figure 4 6

Supplementary Figure 5 7

Supplementary Figure 6 8

Supplementary Figure 7 9

Supplementary Figure 8 10

Supplementary Figure 9 11

Supplementary Figure 10 12

Supplementary Figure 11 13

Supplementary Figure 12 14

Supplementary Figure 13 15

Supplementary Figure 14 16

Supplementary Figure 15 17

Supplementary Table 1 18

Supplementary Table 2 19

Supplementary Table 3 20

Supplementary Table 4 21

Supplementary Table 5 23

References 24

Supplementary Figure 1

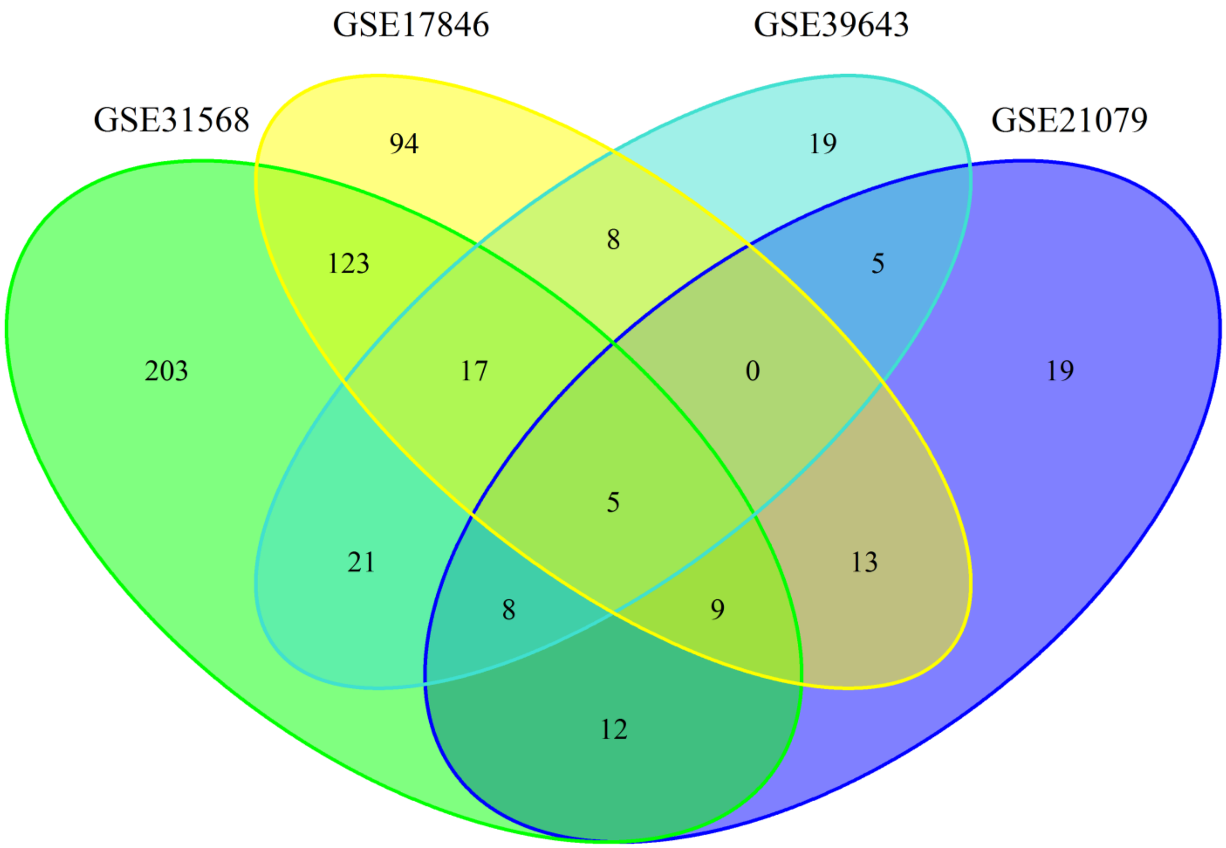

Supplementary Figure 1: Venn diagram of overlapping differentially expressed microRNAs. This figure depicts the overlap of the differentially expressed microRNAs (miRNAs) with a p-value<0.05 between the different miRNA microarray datasets. The direction in which the miRNAs were regulated was not taken into consideration to construct this Venn diagram.

Supplementary Figure 2

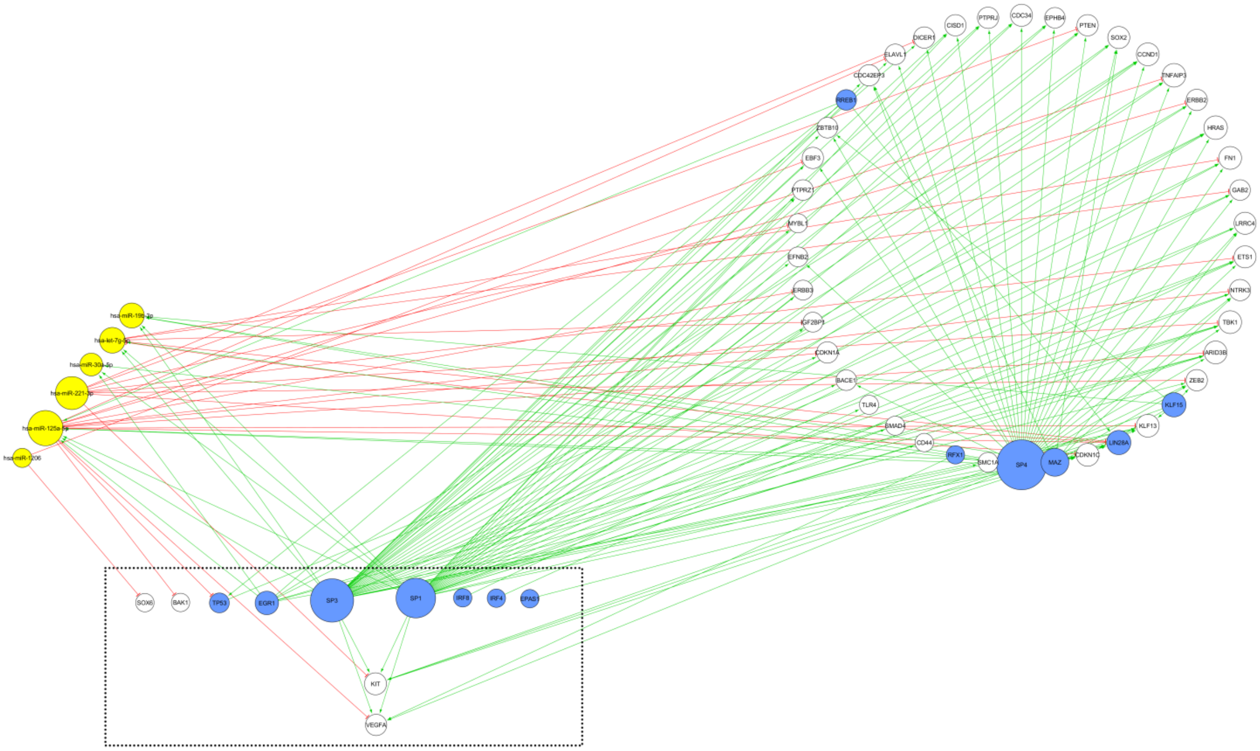

Supplementary Figure 2: Subnetwork created from GO:0048534: Hemopoietic or lymphoid organ development. The nodes contributing to this enriched GO term are depicted in the dashed box. The microRNAs (miRNAs) associated to these enriched nodes are depicted in yellow on the left hand side of the diagram and the remaining genes associated to the enriched nodes have been circularly arranged on the right hand side. All edges (activating edges in green and repressing ones in red) that were present in the original miRNA-based network between the currently depicted nodes are also present in this subnetwork. The nodes in blue represent transcription factors present in the original miRNA-based network. The greater the size of the node is, the greater the degree of the node is in the original miRNA-based network. The nodes that are white are nodes that are neither miRNAs nor transcription factors in the original miRNA-based network. See **Figure 3** for the complete miRNA-based network.

Supplementary Figure 3

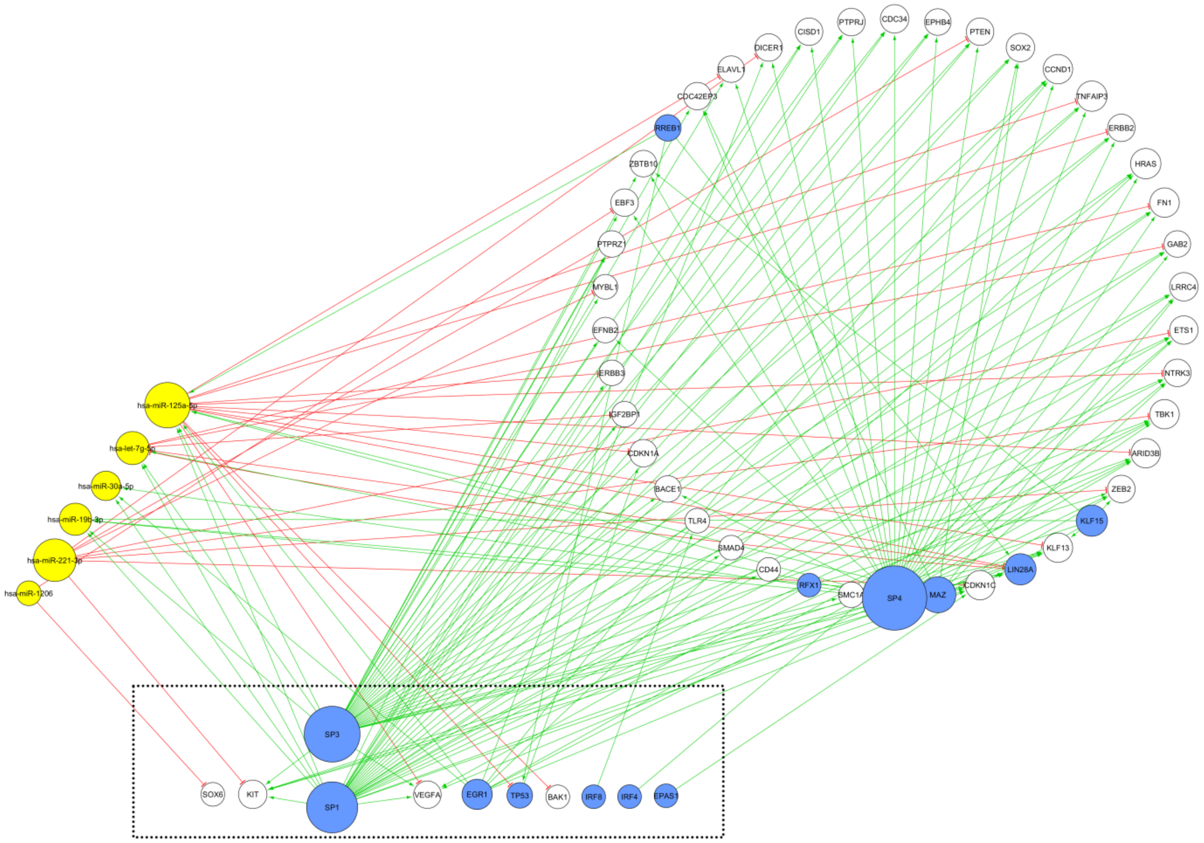

Supplementary Figure 3: Subnetwork created from GO:0002520: Immune system development. The nodes contributing to this enriched GO term are depicted in the dashed box. The microRNAs (miRNAs) associated to these enriched nodes are depicted in yellow on the left hand side of the diagram and the remaining genes associated to the enriched nodes have been circularly arranged on the right hand side. All edges (activating edges in green and repressing ones in red) that were present in the original miRNA-based network between the currently depicted nodes are also present in this subnetwork. The nodes in blue represent transcription factors present in the original miRNA-based network. The greater the size of the node is, the greater the degree of the node is in the original miRNA-based network. The nodes that are white are nodes that are neither miRNAs nor transcription factors in the original miRNA-based network. See **Figure 3** for the complete miRNA-based network.

Supplementary Figure 4

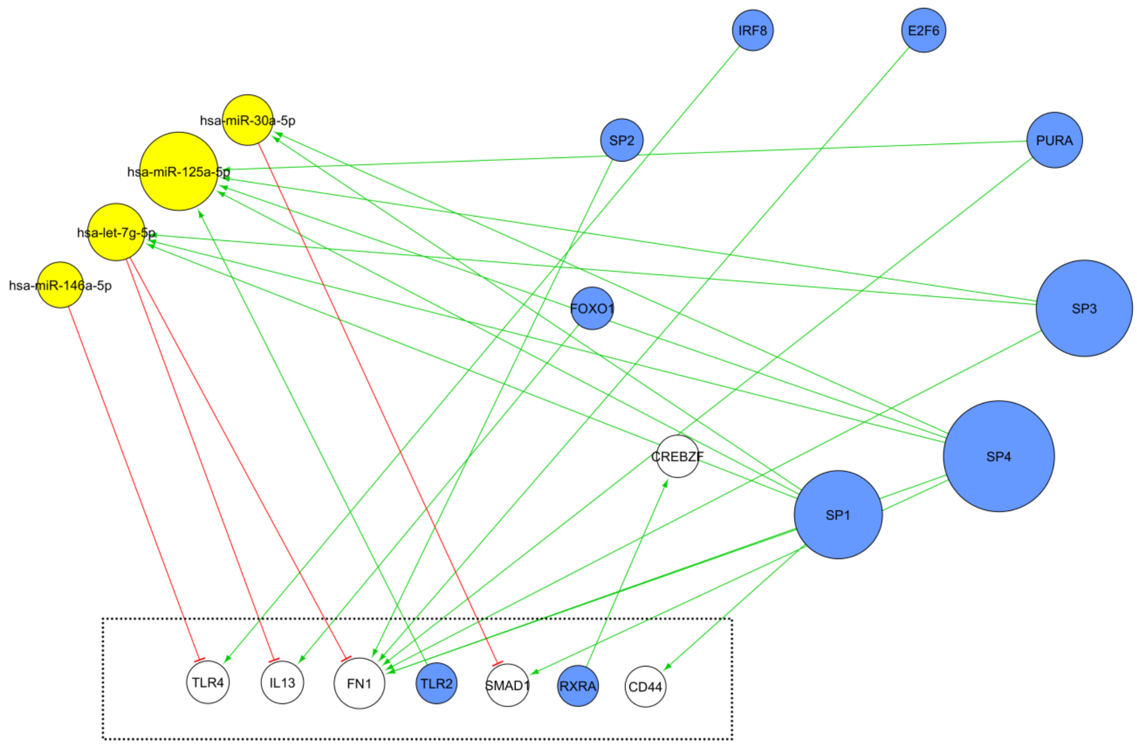

Supplementary Figure 4: Subnetwork created from GO:0006954: Inflammatory response. The nodes contributing to this enriched GO term are depicted in the dashed box. The microRNAs (miRNAs) associated to these enriched nodes are depicted in yellow on the left hand side of the diagram and the remaining genes associated to the enriched nodes have been circularly arranged on the right hand side. All edges (activating edges in green and repressing ones in red) that were present in the original miRNA-based network between the currently depicted nodes are also present in this subnetwork. The nodes in blue represent transcription factors present in the original miRNA-based network. The greater the size of the node is, the greater the degree of the node is in the original miRNA-based network. The nodes that are white are nodes that are neither miRNAs nor transcription factors in the original miRNA-based network. See **Figure 3** for the complete miRNA-based network.

Supplementary Figure 5

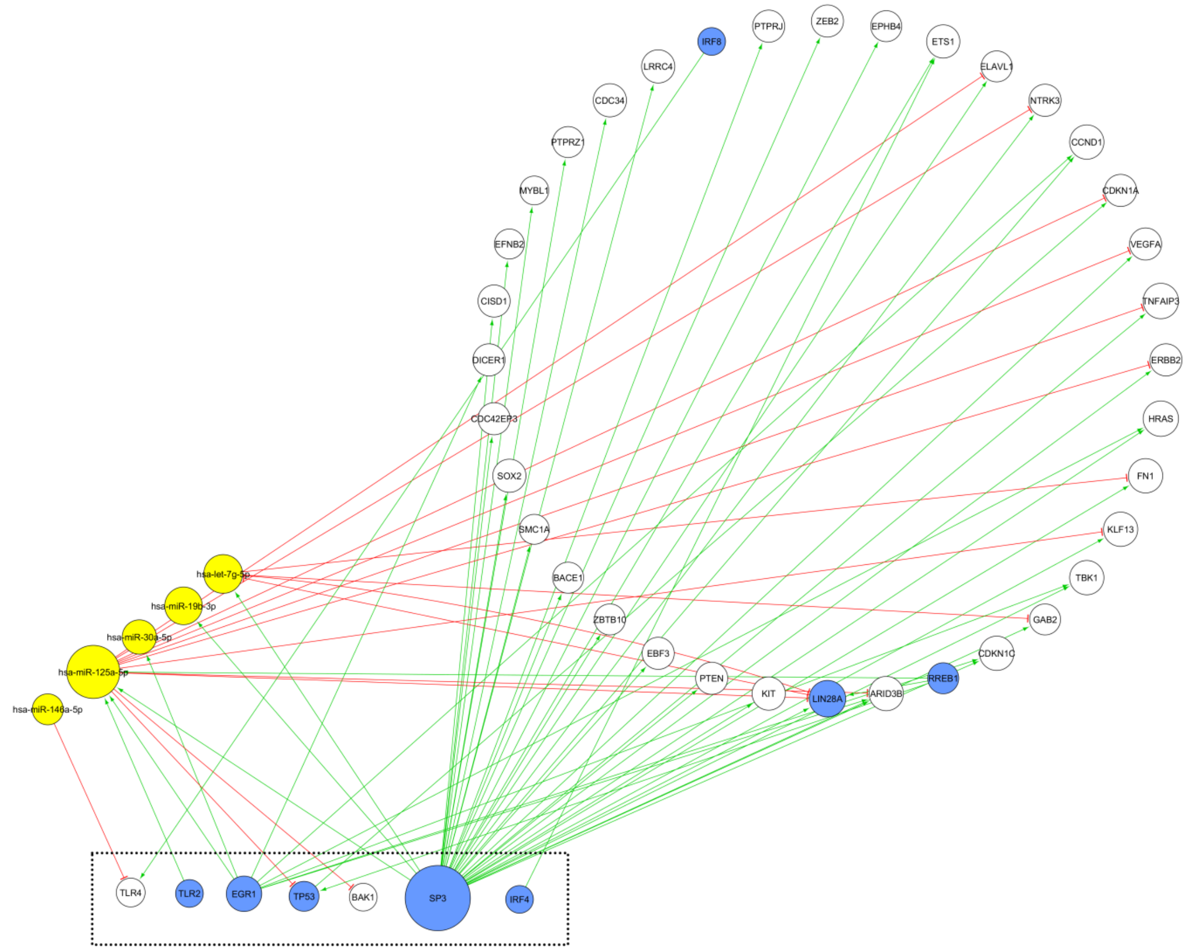

Supplementary Figure 5: Subnetwork created from GO:0045321: Leukocyte activation. The nodes contributing to this enriched GO term are depicted in the dashed box. The microRNAs (miRNAs) associated to these enriched nodes are depicted in yellow on the left hand side of the diagram and the remaining genes associated to the enriched nodes have been circularly arranged on the right hand side. All edges (activating edges in green and repressing ones in red) that were present in the original miRNA-based network between the currently depicted nodes are also present in this subnetwork. The nodes in blue represent transcription factors present in the original miRNA-based network. The greater the size of the node is, the greater the degree of the node is in the original miRNA-based network. The nodes that are white are nodes that are neither miRNAs nor transcription factors in the original miRNA-based network. See **Figure 3** for the complete miRNA-based network.

Supplementary Figure 6

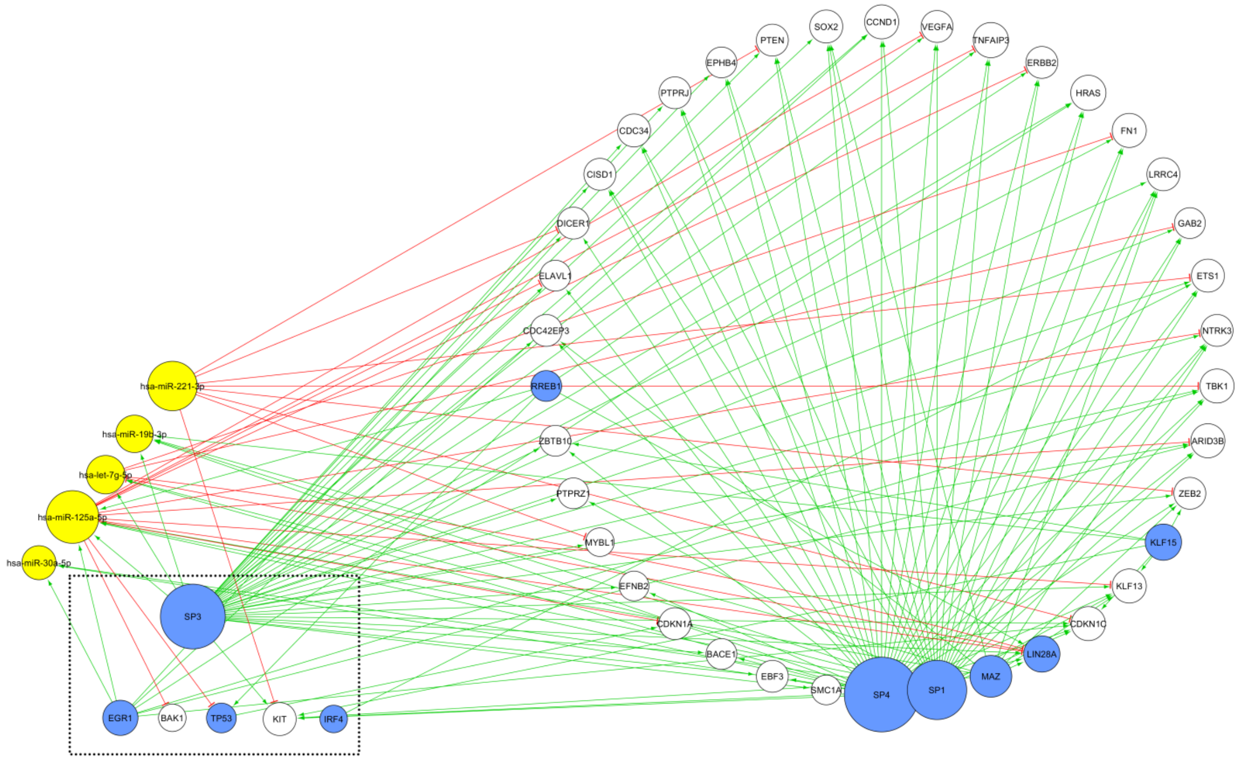

Supplementary Figure 6: Subnetwork created from GO:0002521: Leukocyte differentiation. The nodes contributing to this enriched GO term are depicted in the dashed box. The microRNAs (miRNAs) associated to these enriched nodes are depicted in yellow on the left hand side of the diagram and the remaining genes associated to the enriched nodes have been circularly arranged on the right hand side. All edges (activating edges in green and repressing ones in red) that were present in the original miRNA-based network between the currently depicted nodes are also present in this subnetwork. The nodes in blue represent transcription factors present in the original miRNA-based network. The greater the size of the node is, the greater the degree of the node is in the original miRNA-based network. The nodes that are white are nodes that are neither miRNAs nor transcription factors in the original miRNA-based network. See **Figure 3** for the complete miRNA-based network.

Supplementary Figure 7

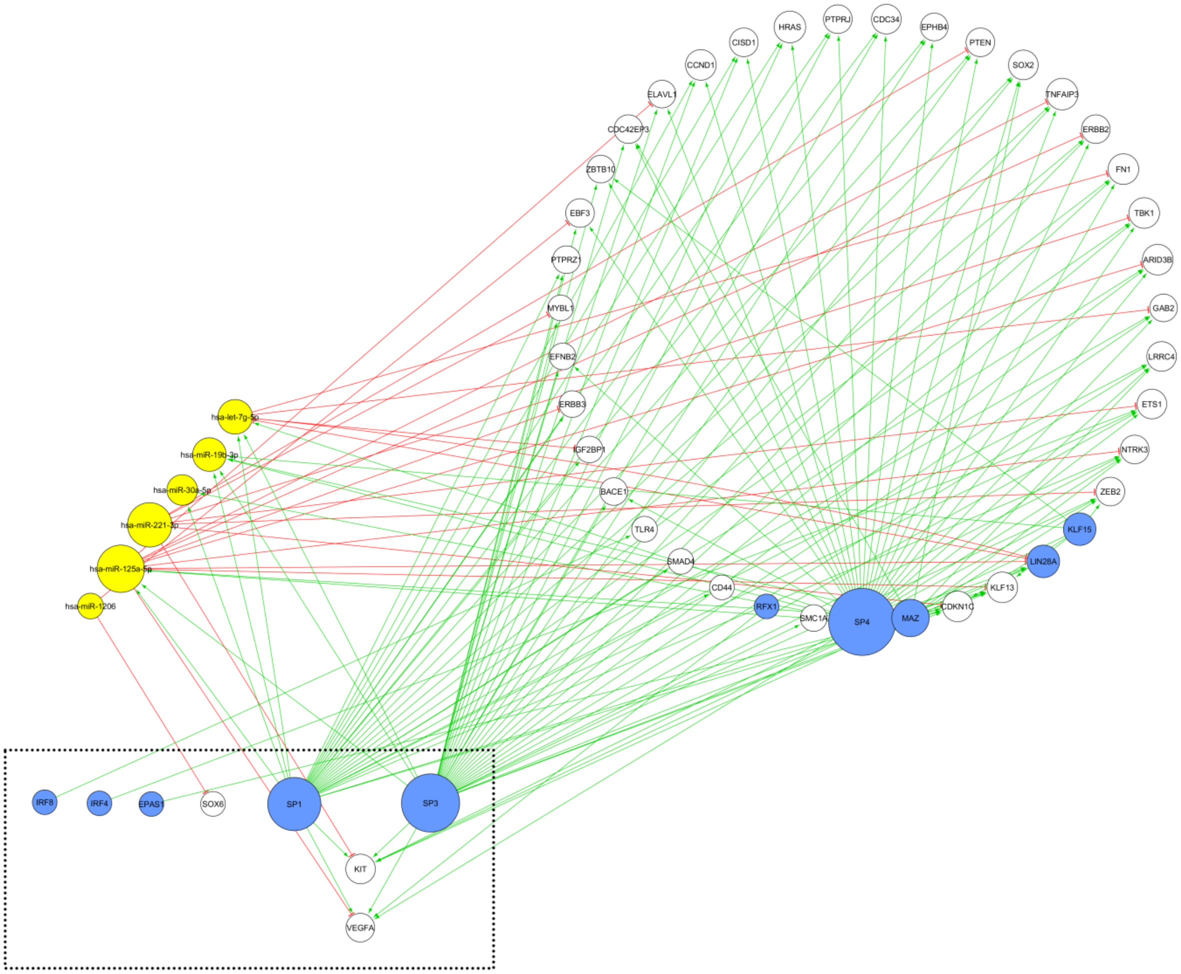

Supplementary Figure 7: Subnetwork created from GO:0030099: Myeloid cell differentiation. The nodes contributing to this enriched GO term are depicted in the dashed box. The microRNAs (miRNAs) associated to these enriched nodes are depicted in yellow on the left hand side of the diagram and the remaining genes associated to the enriched nodes have been circularly arranged on the right hand side. All edges (activating edges in green and repressing ones in red) that were present in the original miRNA-based network between the currently depicted nodes are also present in this subnetwork. The nodes in blue represent transcription factors present in the original miRNA-based network. The greater the size of the node is, the greater the degree of the node is in the original miRNA-based network. The nodes that are white are nodes that are neither miRNAs nor transcription factors in the original miRNA-based network. See **Figure 3** for the complete miRNA-based network.

Supplementary Figure 8

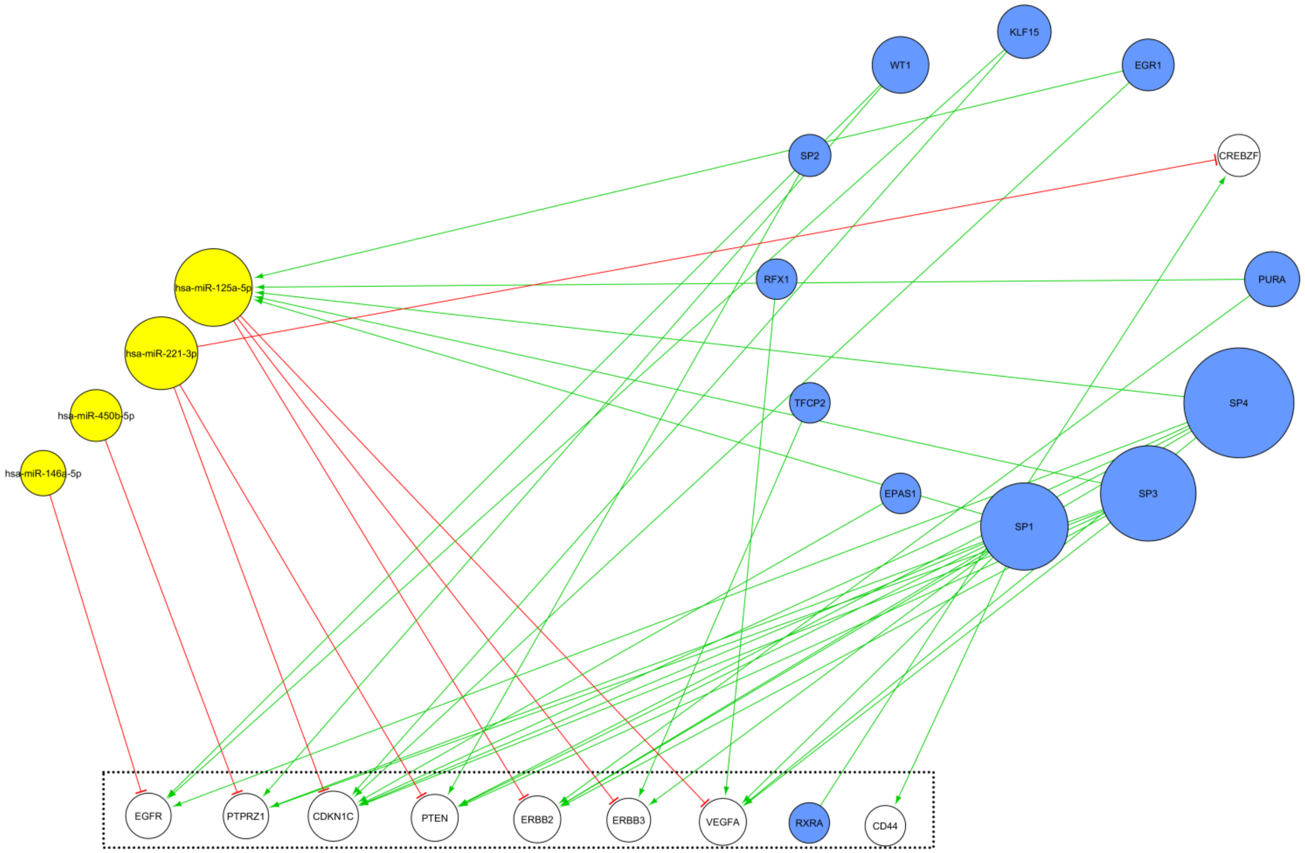

Supplementary Figure 8: Subnetwork created from GO:0048666: Neuron development. The nodes contributing to this enriched GO term are depicted in the dashed box. The microRNAs (miRNAs) associated to these enriched nodes are depicted in yellow on the left hand side of the diagram and the remaining genes associated to the enriched nodes have been circularly arranged on the right hand side. All edges (activating edges in green and repressing ones in red) that were present in the original miRNA-based network between the currently depicted nodes are also present in this subnetwork. The nodes in blue represent transcription factors present in the original miRNA-based network. The greater the size of the node is, the greater the degree of the node is in the original miRNA-based network. The nodes that are white are nodes that are neither miRNAs nor transcription factors in the original miRNA-based network. See **Figure 3** for the complete miRNA-based network.

Supplementary Figure 9

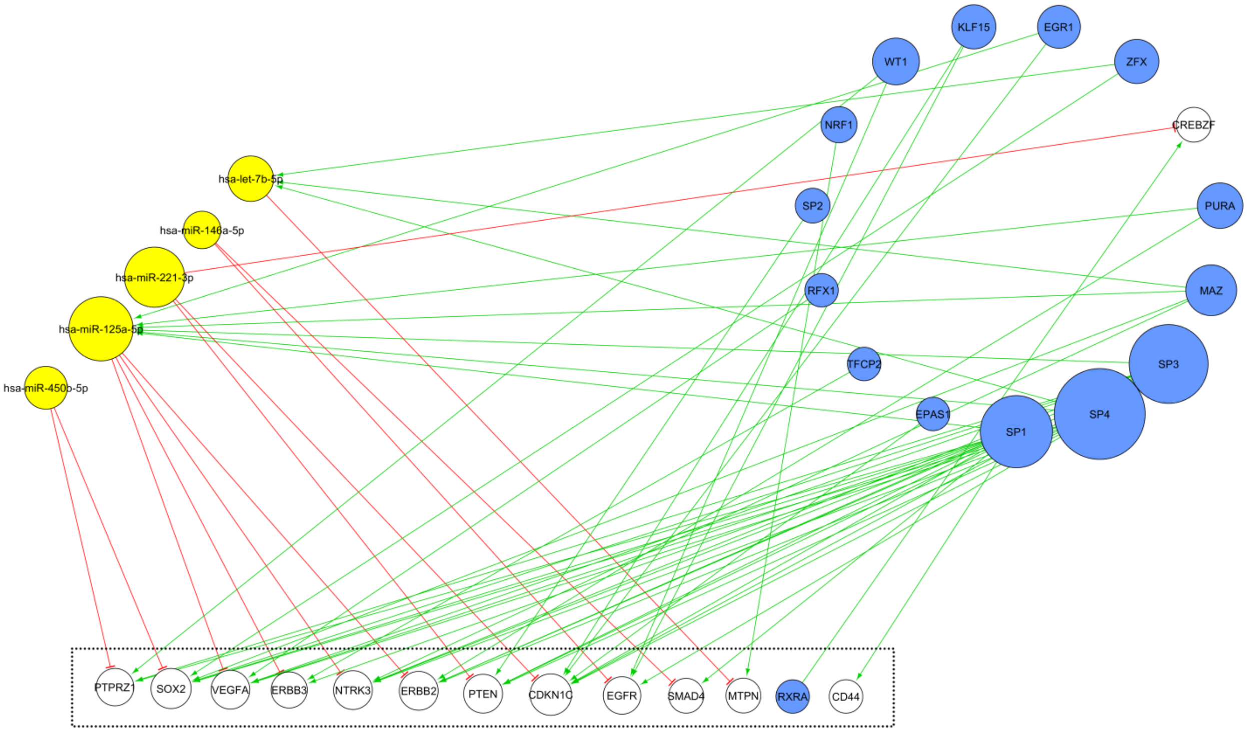

Supplementary Figure 9: Subnetwork created from GO:0030182: Neuron differentiation. The nodes contributing to this enriched GO term are depicted in the dashed box. The microRNAs (miRNAs) associated to these enriched nodes are depicted in yellow on the left hand side of the diagram and the remaining genes associated to the enriched nodes have been circularly arranged on the right hand side. All edges (activating edges in green and repressing ones in red) that were present in the original miRNA-based network between the currently depicted nodes are also present in this subnetwork. The nodes in blue represent transcription factors present in the original miRNA-based network. The greater the size of the node is, the greater the degree of the node is in the original miRNA-based network. The nodes that are white are nodes that are neither miRNAs nor transcription factors in the original miRNA-based network. See **Figure 3** for the complete miRNA-based network.

Supplementary Figure 10

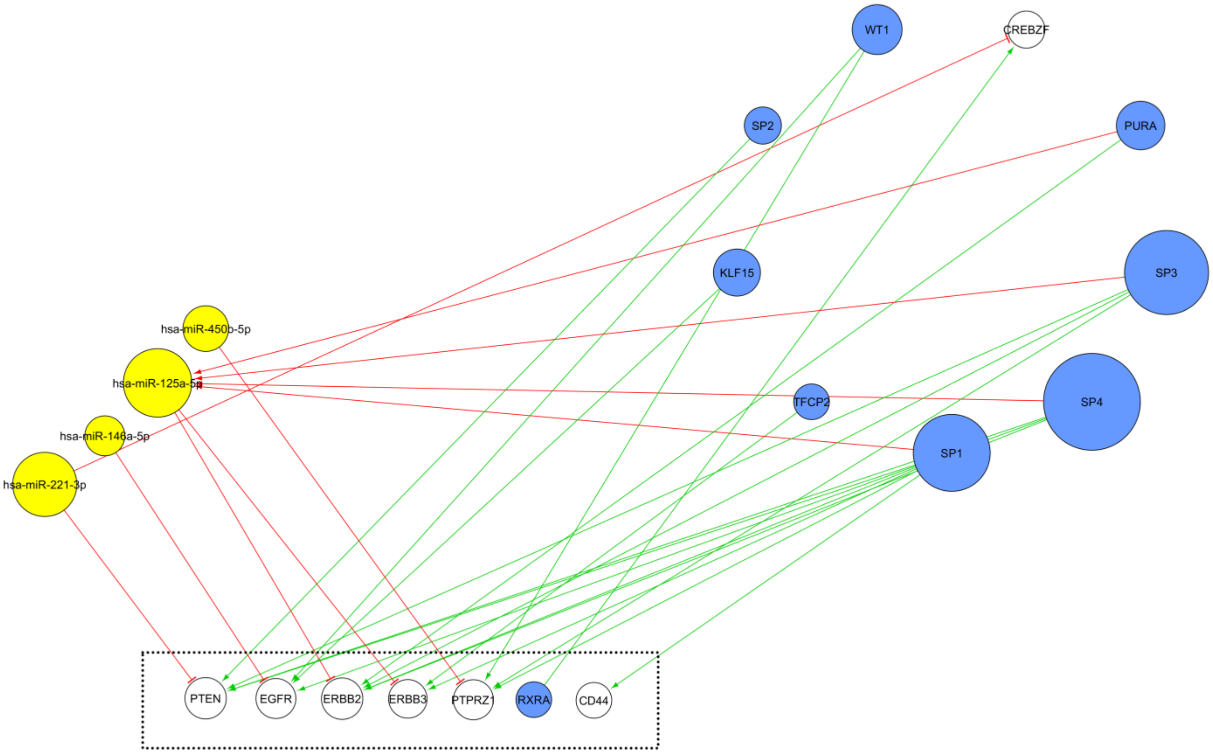

Supplementary Figure 10: Subnetwork created from GO:0031175: Neuron projection development. The nodes contributing to this enriched GO term are depicted in the dashed box. The microRNAs (miRNAs) associated to these enriched nodes are depicted in yellow on the left hand side of the diagram and the remaining genes associated to the enriched nodes have been circularly arranged on the right hand side. All edges (activating edges in green and repressing ones in red) that were present in the original miRNA-based network between the currently depicted nodes are also present in this subnetwork. The nodes in blue represent transcription factors present in the original miRNA-based network. The greater the size of the node is, the greater the degree of the node is in the original miRNA-based network. The nodes that are white are nodes that are neither miRNAs nor transcription factors in the original miRNA-based network. See **Figure 3** for the complete miRNA-based network.

Supplementary Figure 11

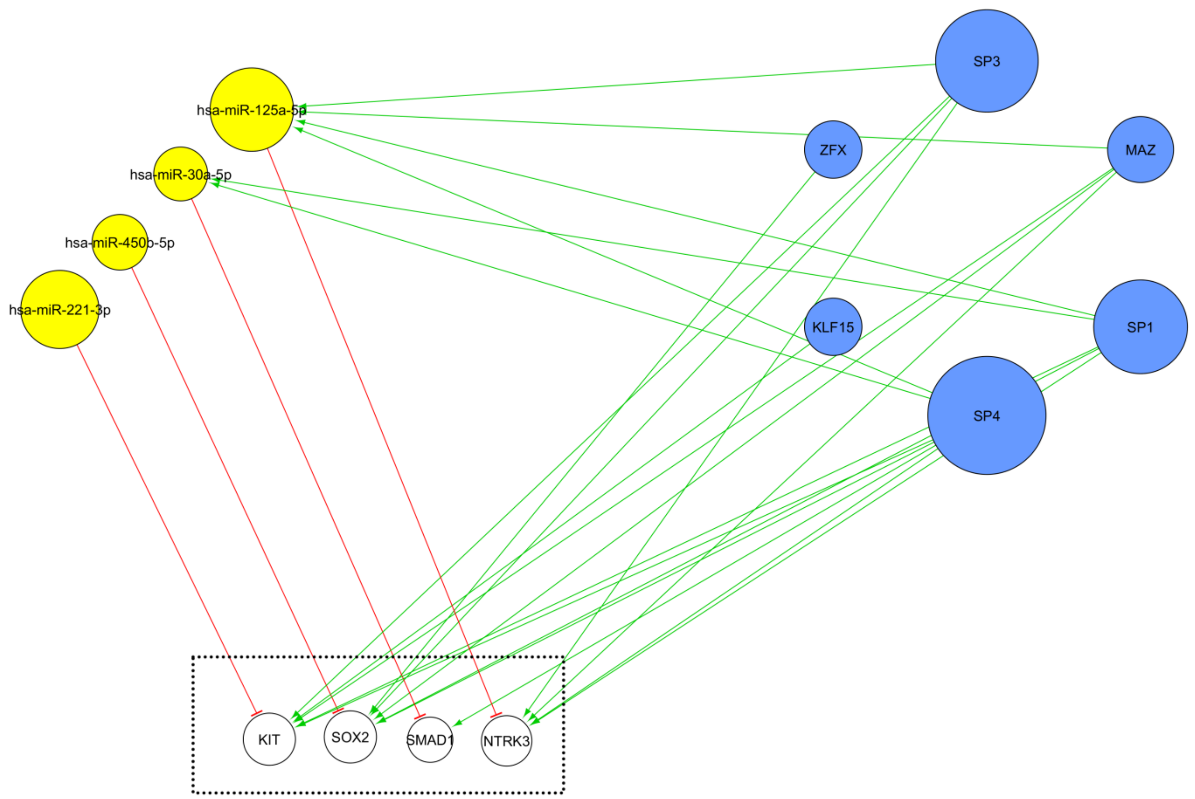

Supplementary Figure 11: Subnetwork created from GO:0050769: Positive regulation of neurogenesis. The nodes contributing to this enriched GO term are depicted in the dashed box. The microRNAs (miRNAs) associated to these enriched nodes are depicted in yellow on the left hand side of the diagram and the remaining genes associated to the enriched nodes have been circularly arranged on the right hand side. All edges (activating edges in green and repressing ones in red) that were present in the original miRNA-based network between the currently depicted nodes are also present in this subnetwork. The nodes in blue represent transcription factors present in the original miRNA-based network. The greater the size of the node is, the greater the degree of the node is in the original miRNA-based network. The nodes that are white are nodes that are neither miRNAs nor transcription factors in the original miRNA-based network. See **Figure 3** for the complete miRNA-based network.

Supplementary Figure 12

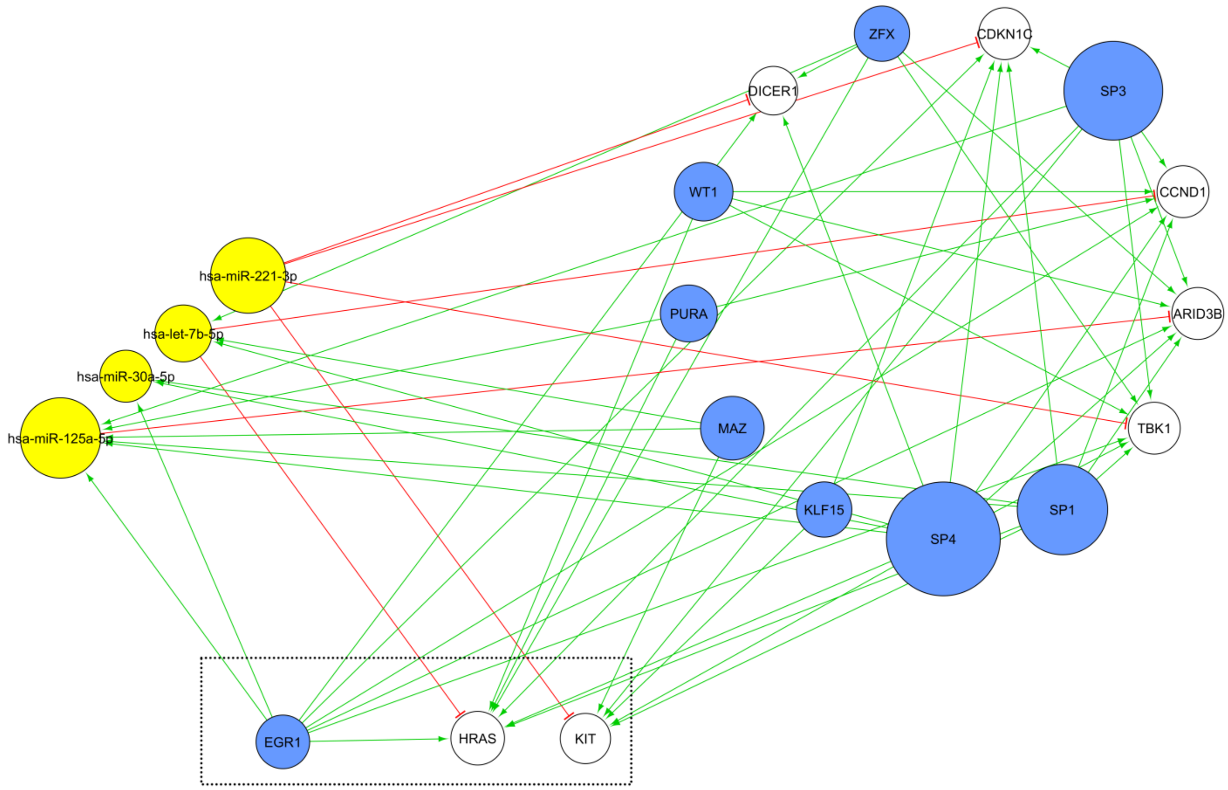

Supplementary Figure 12: Subnetwork created from GO:0048169: Regulation of long-term neuronal synaptic plasticity. The nodes contributing to this enriched GO term are depicted in the dashed box. The microRNAs (miRNAs) associated to these enriched nodes are depicted in yellow on the left hand side of the diagram and the remaining genes associated to the enriched nodes have been circularly arranged on the right hand side. All edges (activating edges in green and repressing ones in red) that were present in the original miRNA-based network between the currently depicted nodes are also present in this subnetwork. The nodes in blue represent transcription factors present in the original miRNA-based network. The greater the size of the node is, the greater the degree of the node is in the original miRNA-based network. The nodes that are white are nodes that are neither miRNAs nor transcription factors in the original miRNA-based network. See **Figure 3** for the complete miRNA-based network.

Supplementary Figure 13

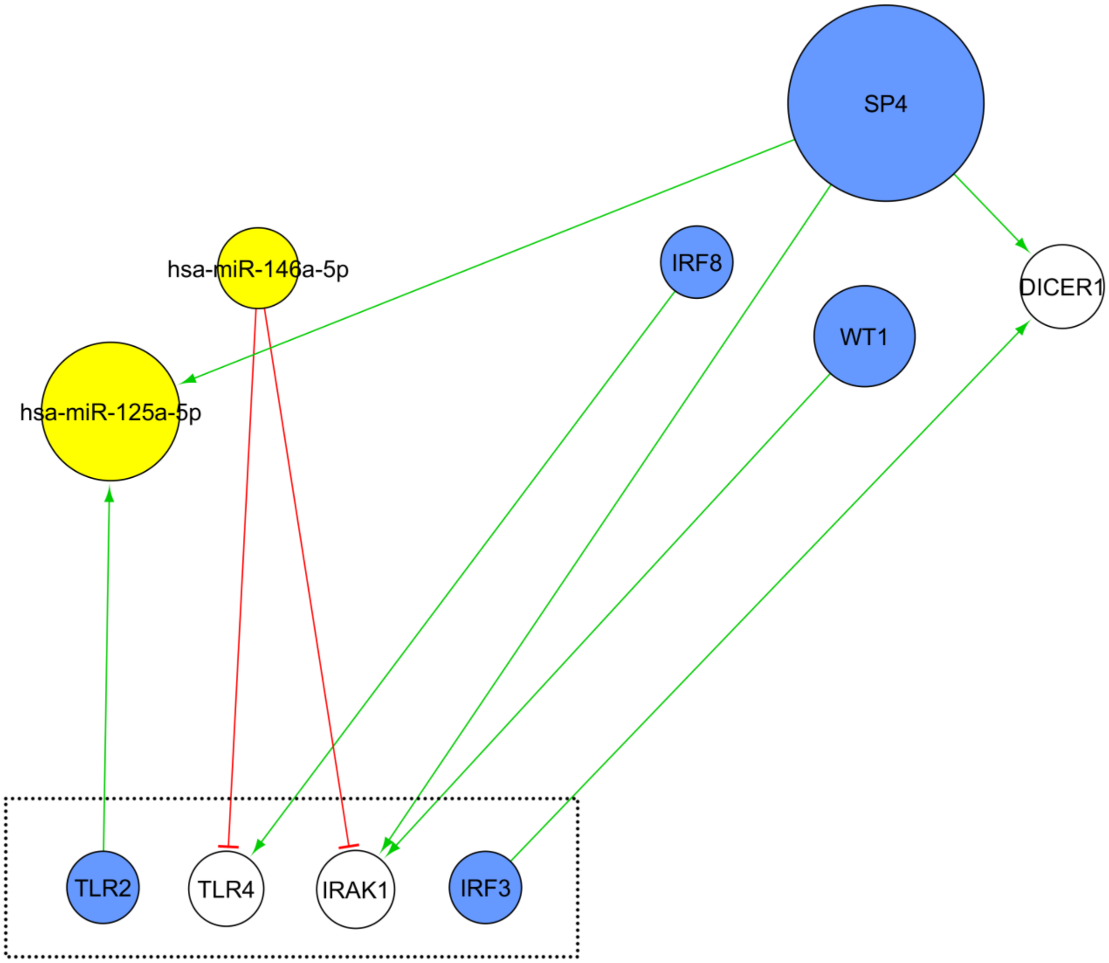

Supplementary Figure 13: Subnetwork created from GO:0002237: Response to molecule of bacterial origin. The nodes contributing to this enriched GO term are depicted in the dashed box. The microRNAs (miRNAs) associated to these enriched nodes are depicted in yellow on the left hand side of the diagram and the remaining genes associated to the enriched nodes have been circularly arranged on the right hand side. All edges (activating edges in green and repressing ones in red) that were present in the original miRNA-based network between the currently depicted nodes are also present in this subnetwork. The nodes in blue represent transcription factors present in the original miRNA-based network. The greater the size of the node is, the greater the degree of the node is in the original miRNA-based network. The nodes that are white are nodes that are neither miRNAs nor transcription factors in the original miRNA-based network. See **Figure 3** for the complete miRNA-based network.

Supplementary Figure 14

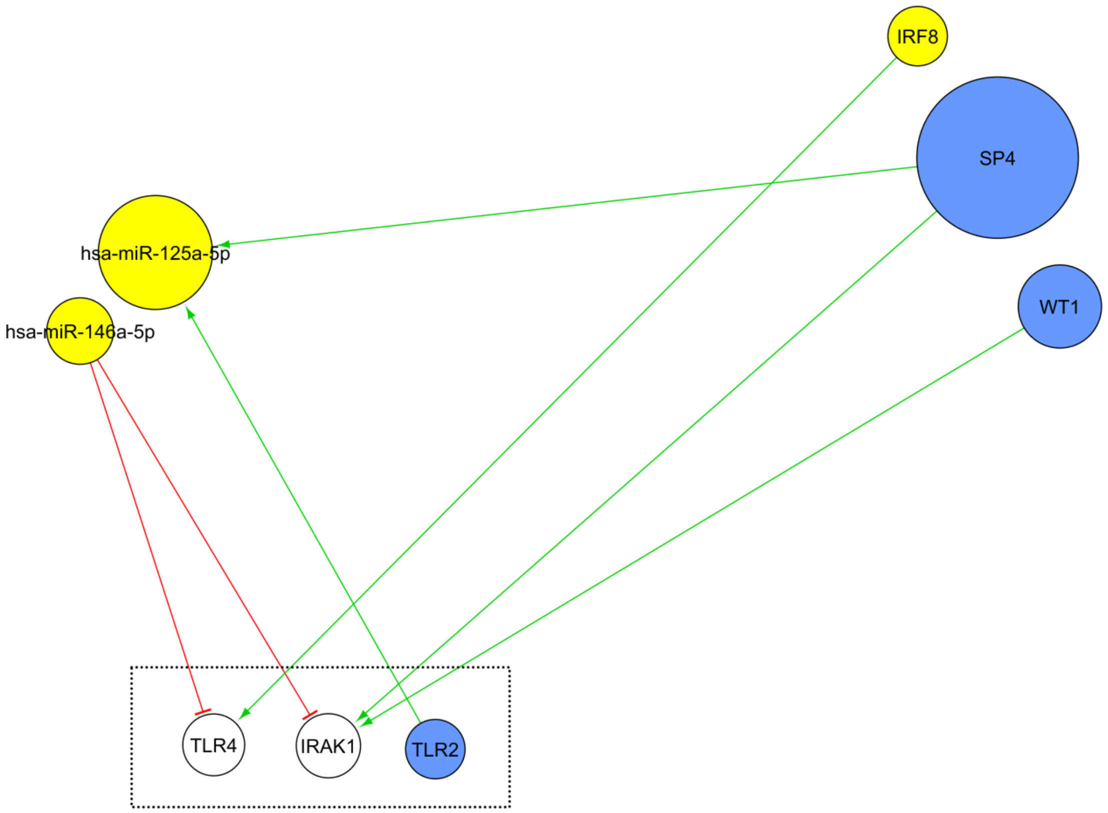

Supplementary Figure 14: Subnetwork created from either GO:0002218: Activation of innate immune response, from GO:0002758: Innate immune response- activating signal transduction, or from GO:0002224:Toll-like receptor signaling pathway. These three GO terms are associated to the same enriched nodes: TLR2, TLR4, and IRAK1. The nodes contributing to this enriched GO term are depicted in the dashed box. The microRNAs (miRNAs) associated to these enriched nodes are depicted in yellow on the left hand side of the diagram and the remaining genes associated to the enriched nodes have been circularly arranged on the right hand side. All edges (activating edges in green and repressing ones in red) that were present in the original miRNA-based network between the currently depicted nodes are also present in this subnetwork. The nodes in blue represent transcription factors present in the original miRNA-based network. The greater the size of the node is, the greater the degree of the node is in the original miRNA-based network. The nodes that are white are nodes that are neither miRNAs nor transcription factors in the original miRNA-based network. See **Figure 3** for the complete miRNA-based network.

Supplementary Figure 15

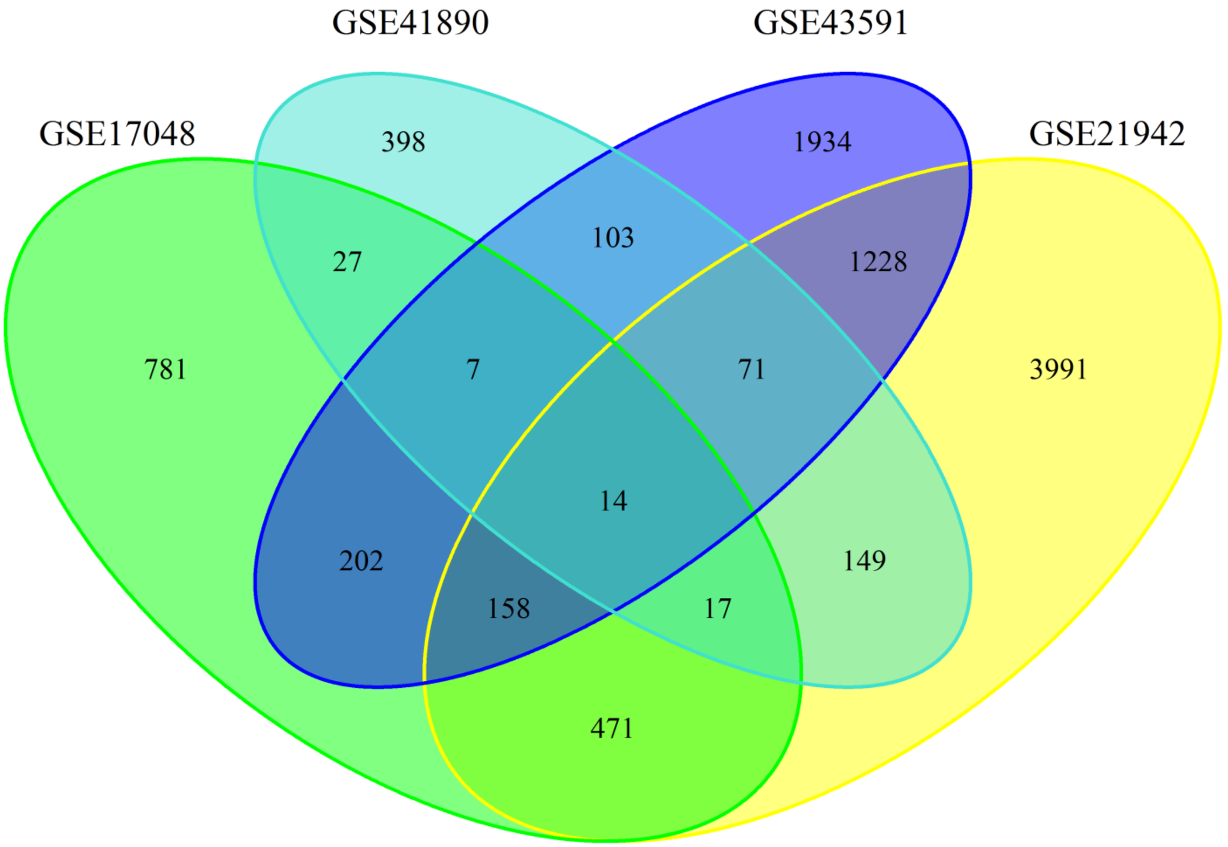

Supplementary Figure 15: Venn diagram of overlapping differentially expressed protein-coding genes with a p-value<0.05 per dataset. This figure depicts the overlap of the differentially expressed genes with a p-value<0.05 between the different. The direction in which the genes were regulated was not taken into consideration to construct this Venn diagram.

Supplementary Table 1

miRNA	Target	miRNA	Target
let-7b-5p	CCND1	miR-125a-5p	NTRK3
let-7b-5p	CDC34	miR-125a-5p	TNFAIP3
let-7b-5p	HRAS	miR-125a-5p	TP53
let-7b-5p	IGF2BP1	miR-125a-5p	VEGFA
let-7b-5p	LIN28A	miR-146a-5p	EGFR
let-7b-5p	MTPN	miR-146a-5p	IRAK1
let-7g-5p	FN1	miR-146a-5p	SMAD4
let-7g-5p	GAB2	miR-146a-5p	TLR4
let-7g-5p	HMGA2	miR-221-3p	BNIP3L
let-7g-5p	IGF2BP1	miR-221-3p	CDKN1B
let-7g-5p	IL13	miR-221-3p	CDKN1C
miR-19b-3p	CUL5	miR-221-3p	CREBZF
miR-19b-3p	MYCN	miR-221-3p	DDIT4
miR-20b-5p	CDKN1A	miR-221-3p	DICER1
miR-20b-5p	EFNB2	miR-221-3p	DIRAS3
miR-20b-5p	EPHB4	miR-221-3p	DKK2
miR-30a-5p	BECN1	miR-221-3p	ETS1
miR-30a-5p	DTL	miR-221-3p	KIT
miR-30a-5p	RUNX2	miR-221-3p	MYBL1
miR-30a-5p	SMAD1	miR-221-3p	PSMD9
miR-125a-5p	ARID3B	miR-221-3p	PTEN
miR-125a-5p	BAK1	miR-221-3p	TBK1
miR-125a-5p	CD34	miR-221-3p	TIMP3
miR-125a-5p	CDKN1A	miR-221-3p	ZEB2
miR-125a-5p	ELAVL1	miR-328	ABCG2
miR-125a-5p	ERBB2	miR-328	BACE1
miR-125a-5p	ERBB3	miR-328	CD44
miR-125a-5p	KLF13	miR-328	H2AFX
miR-125a-5p	LIN28A	miR-328	PTPRJ

Supplementary Table 1: Validated microRNA-target pairs that were included in the microRNA-based network. The validated microRNA-target pairs were found using miRTarBase, Release 3.5, and TarBase, Release 6.0, for the microRNA-based network[1,2]. The target pairs were found using the 18 miRNAs that were differentially expressed and dysregulated in the same direction in at least 3 of the 4 microRNA microarray datasets (see **Tables 1 and 2**).

Supplementary Table 2

miRNA	Target	miRNA	Target	miRNA	Target
miR-125a-5p	ENPEP	miR-300	TRIM63	miR-450b-5p	SOX2
miR-300	ANO4	miR-300	VASH2	miR-450b-5p	UBR1
miR-300	DCAF12	miR-450b-5p	ATP11C	miR-450b-5p	ZBTB10
miR-300	LCORL	miR-450b-5p	CDC42EP3	miR-580	CISD1
miR-300	LRRC4	miR-450b-5p	ELAVL4	miR-580	FGF7
miR-300	NR5A2	miR-450b-5p	PTPRZ1	miR-1206	EBF3
miR-300	PAN3	miR-450b-5p	SMC1A	miR-1206	SOX6

Supplementary Table 2: Predicted miRNA-target pairs that were included in the miRNA-based network. The predicted miRNA-target pairs were found using TargetScan, Release 6.2, miRDB, Release 4.0, and microT-CDS, Release 5.0[3–5]. The target pairs were found using the 18 miRNAs that were differentially expressed and dysregulated in the same direction in at least 3 of the 4 microRNA microarray datasets (see **Tables 1 and 2**).

Supplementary Table 3

TF	miRNA	TF	miRNA
E2F6	miR-19b-3p	SP1	miR-19b-3p
EGR4	miR-125a-5p	SP1	miR-30a-5p
GABPA	miR-328	SP3	let-7g-5p
KLF15	miR-19b-3p	SP3	miR-19b-3p
KLF15	miR-20b-5p	SP3	miR-125a-5p
MAZ	miR-125a-5p	SP4	let-7g-5p
MAZ	let-7b-5p	SP4	miR-125a-5p
MAZ	miR-19b-3p	SP4	let-7b-5p
PLAG1	miR-125a-5p	SP4	miR-19b-3p
PURA	miR-125a-5p	SP4	miR-30a-5p
RREB1	miR-125a-5p	TEF	miR-221-3p
SP1	let-7g-5p	ZFX	let-7b-5p
SP1	miR-125a-5p		

Supplementary Table 3: Predicted transcription factor and miRNA interaction pairs found using FIMO[6] in combination with HOCOMOCO[7]. FIMO[6] and HOCOMOCO[7] were used in order to find predicted miRNA targets of the transcription factors (TFs) in HOCOMOCO[7]. The target pairs were found using the 18 miRNAs that were differentially expressed and dysregulated in the same direction in at least 3 of the 4 microRNA microarray datasets (see **Tables 1 and 2**).

Supplementary Table 4

TF	Gene	TF	Gene	TF	Gene
ARNT2	ABCG2	PURA	ERBB2	SP3	EPHB4
CTCF	CUL5	PURA	FN1	SP3	ERBB2
CTCF	TNFAIP3	PURA	HRAS	SP3	ESR1
E2F4	SRSF1	PURA	LCORL	SP3	ETS1
E2F6	ETS1	PURA	LIN28A	SP3	FN1
E2F6	FN1	PURA	LRRC4	SP3	GAB2
EGR1	ARID3B	RFX1	VEGFA	SP3	HRAS
EGR1	CCND1	RREB1	DCAF12	SP3	KIT
EGR1	CDKN1C	RREB1	LIN28A	SP3	KLF13
EGR1	DICER1	RREB1	TP53	SP3	LIN28A
EGR1	HRAS	RXRA	CREBZF	SP3	LRRC4
EGR1	TBK1	SP1	ARID3B	SP3	MYBL1
EGR4	EBF3	SP1	CCND1	SP3	NTRK3
ELK3	NR5A2	SP1	CD44	SP3	PTEN
EPAS1	CDKN1C	SP1	CDC34	SP3	PTPRJ
FOS	LIN28A	SP1	CDKN1C	SP3	PTPRZ1
FOXO1	CDKN1A	SP1	CISD1	SP3	SMC1A
FOXO1	IL13	SP1	EPHB4	SP3	SOX2
IRF3	DICER1	SP1	ERBB2	SP3	TBK1
IRF4	ETS1	SP1	ERBB3	SP3	TNFAIP3
IRF8	TLR4	SP1	ESR1	SP3	VEGFA
KLF15	CDKN1C	SP1	ETS1	SP3	ZBTB10
KLF15	DCAF12	SP1	FN1	SP3	ZEB2
KLF15	EGFR	SP1	GAB2	SP4	ARID3B
KLF15	KIT	SP1	HRAS	SP4	BACE1
KLF15	KLF13	SP1	IGF2BP1	SP4	CCND1
KLF15	ZBTB10	SP1	KIT	SP4	CDC34
KLF15	ZEB2	SP1	KLF13	SP4	CDC42EP3
MAZ	CDC42EP3	SP1	LIN28A	SP4	CDKN1B
MAZ	ELAVL4	SP1	LRRC4	SP4	CDKN1C
MAZ	KIT	SP1	NTRK3	SP4	CISD1
MAZ	KLF13	SP1	PTEN	SP4	DCAF12
MAZ	LCORL	SP1	PTPRJ	SP4	DICER1
MAZ	LIN28A	SP1	PTPRZ1	SP4	EBF3
MAZ	LRRC4	SP1	SMAD4	SP4	EFNB2
MAZ	NTRK3	SP1	SOX2	SP4	EGFR
MAZ	PAN3	SP1	TBK1	SP4	ELAVL1
MAZ	SOX2	SP1	TNFAIP3	SP4	EPHB4
MAZ	ZEB2	SP1	VEGFA	SP4	ERBB2
NRF1	BNIP3L	SP2	FN1	SP4	ESR1
NRF1	CDC34	SP2	PTEN	SP4	ETS1
NRF1	MTPN	SP3	ARID3B	SP4	FN1
PAX5	TNFAIP3	SP3	BACE1	SP4	GAB2
PAX8	TNFAIP3	SP3	CCND1	SP4	HRAS
PLAG1	ABCG2	SP3	CDC34	SP4	IRAK1
PPARD	CDC34	SP3	CDC42EP3	SP4	KIT
PRDM1	ATP11C	SP3	CDKN1C	SP4	KLF13
PURA	BACE1	SP3	CISD1	SP4	LIN28A
PURA	CCND1	SP3	EBF3	SP4	LRRC4
PURA	CD34	SP3	EFNB2	SP4	MYCN
PURA	CDC42EP3	SP3	ELAVL1	SP4	NR5A2

SP4	NTRK3	TBX2	CDKN1A	WT1	PTPRJ
SP4	PAN3	TBX2	SMC1A	WT1	PTPRZ1
SP4	PTEN	TBX2	TRIM63	WT1	TBK1
SP4	PTPRJ	TFCP2	ERBB3	ZFX	ARID3B
SP4	RUNX2	TP53	CDKN1A	ZFX	DICER1
SP4	SMAD1	WT1	ARID3B	ZFX	EBF3
SP4	SOX2	WT1	CCND1	ZFX	ELAVL1
SP4	TBK1	WT1	CISD1	ZFX	HRAS
SP4	TNFAIP3	WT1	EGFR	ZFX	SOX2
SP4	VEGFA	WT1	HRAS	ZFX	TBK1
SP4	ZBTB10	WT1	IRAK1	ZFX	TNFAIP3
SP4	ZEB2	WT1	KLF13		
STAT4	MYCN	WT1	MYCN		

Supplementary Table 4: Predicted transcription factor and gene interaction pairs found using FIMO[6] in combination with HOCOMOCO[7]. FIMO[6] and HOCOMOCO[7] were used in order to find predicted targets of the transcription factors (TFs) in HOCOMOCO[7] for the microRNA-based network.

Supplementary Table 5

AAMP	DDOST	MED9	RAP2B
ABHD15	DDX50	MESDC1	RBBP6
ADRB2	DEDD2	METTL5	REPS1
AKT3	DUSP12	MFAP1	RGS9
ALG2	DYNLL2	MIP	RGS9BP
ARAP1	EIF2AK2	MIPEP	RRAD
**BEX1**	EIF3M	MRPL1	SIGLEC17P
**BEX2**	EIF4A2	MRPL27	SLC25A21
BLCAP	EIF4EBP2	MRPL41	SLC39A14
BRIX1	EIF4G1	MRPS27	SNRPA1
BUB1	ELF4	NAA30	SOAT1
C19orf43	ELMO2	NCOA6	SRP72
CA5B	EMC1	NCOR1	SRPRB
CAMK2N1	ERCC4	NDC80	SWT1
CASC3	EXT2	NLRP3	TBC1D20
CASP9	EXT3	NOP58	TES
CCDC59	F8A1	NSMCE4A	TMEM107
CD244	FAM122A	ODC1	TNPO1
CD69	GALNT6	ORC3	TOP2A
CDK2AP2	GIGYF2	**PALLD**	TOR1AIP1
CERS6	GPBP1	PAPOLG	TSEN34
CHSY1	HNRNPH3	PLEKHF1	TUBB
CLIC3	HSPA1A	PMAIP1	UBE2G1
CORO1A	ICAM3	POLDIP3	UPF1
CRBN	IFNAR1	PPP1CA	UXT
CSTF2T	IFT74	PPP1R18	VKORC1L1
CTDSP2	KRR1	PPWD1	VPS37C
CXCR3	LARP7	PRDX5	VRK1
DCAF4	LPCAT1	RAB21	YWHAG
DCAF7	MAFIP	RAB5B	ZBTB34
DCTN6	MAPK1	RAC1	**ZNF264**
DDIT3	MATR3	RAD1	ZNF777

Supplementary Table 5: Differentially expressed genes. 128 genes that were differentially expressed in at least 3 of the 4 protein-coding microarrays and that were regulated in the same direction (**Table 1**). Genes in bold represent genes that were differentially expressed and regulated in the same direction in all four protein-coding microarray datasets.

References

1. Hsu, S.-D. *et al.* miRTarBase: a database curates experimentally validated microRNA-target interactions. *Nucleic Acids Res.* **39,** D163–D169 (2011).
2. Vergoulis, T. *et al.* TarBase 6.0: capturing the exponential growth of miRNA targets with experimental support. *Nucleic Acids Res.* **40,** D222–D229 (2012).
3. Grimson, A. *et al.* MicroRNA targeting specificity in mammals: determinants beyond seed pairing. *Mol. Cell* **27,** 91–105 (2007)
4. Wang, X. & El Naqa, I. M. Prediction of both conserved and nonconserved microRNA targets in animals. *Bioinformatics* **24,** 325–332 (2008).
5. Paraskevopoulou, M. D. *et al.* DIANA-microT web server v5.0: service integration into miRNA functional analysis workflows. *Nucleic Acids Res.* **41,** W169–W173 (2013).
6. Grant, C. E., Bailey, T. L. & Noble, W. S. FIMO: scanning for occurrences of a given motif. *Bioinformatics* **27,** 1017–1018 (2011).
7. Kulakovskiy, I. V *et al.* HOCOMOCO: a comprehensive collection of human transcription factor binding sites models. *Nucleic Acids Res.* **41,** D195–D202 (2013).
